# Structural and evolutionary analyses of the *Plasmodium falciparum* chloroquine resistance transporter

**DOI:** 10.1038/s41598-020-61181-1

**Published:** 2020-03-16

**Authors:** Romain Coppée, Audrey Sabbagh, Jérôme Clain

**Affiliations:** 1 Université de Paris, UMR 261 MERIT, IRD, F-75006 Paris, France; 2 Institut Universitaire de France (IUF), Paris, France; 3 Centre National de Référence du Paludisme, Hôpital Bichat-Claude Bernard, Assistance Publique des Hôpitaux de Paris, F-75018 Paris, France

**Keywords:** Phylogeny, Protein analysis, Protein function predictions, Protein structure predictions, Molecular evolution

## Abstract

Mutations in the *Plasmodium falciparum* chloroquine resistance transporter (PfCRT) confer resistance to several antimalarial drugs such as chloroquine (CQ) or piperaquine (PPQ), a partner molecule in current artemisinin-based combination therapies. As a member of the Drug/Metabolite Transporter (DMT) superfamily, the vacuolar transporter PfCRT may translocate substrate molecule(s) across the membrane of the digestive vacuole (DV), a lysosome-like organelle. However, the physiological substrate(s), the transport mechanism and the functional regions of PfCRT remain to be fully characterized. Here, we hypothesized that identification of evolutionary conserved sites in a tertiary structural context could help locate putative functional regions of PfCRT. Hence, site-specific substitution rates were estimated over *Plasmodium* evolution at each amino acid sites, and the PfCRT tertiary structure was predicted in both inward-facing (open-to-vacuole) and occluded states through homology modeling using DMT template structures sharing <15% sequence identity with PfCRT. We found that the vacuolar-half and membrane-spanning domain (and especially the transmembrane helix 9) of PfCRT were more conserved, supporting that its physiological substrate is expelled out of the parasite DV. In the PfCRT occluded state, some evolutionary conserved sites, including positions related to drug resistance mutations, participate in a putative binding pocket located at the core of the PfCRT membrane-spanning domain. Through structural comparison with experimentally-characterized DMT transporters, we identified several conserved PfCRT amino acid sites located in this pocket as robust candidates for mediating substrate transport. Finally, *in silico* mutagenesis revealed that drug resistance mutations caused drastic changes in the electrostatic potential of the transporter vacuolar entry and pocket, facilitating the escape of protonated CQ and PPQ from the parasite DV.

## Introduction

Resistance to antimalarial drugs continues to threaten malaria control. Formerly used as first-line treatment for uncomplicated *Plasmodium falciparum* malaria in most endemic areas, chloroquine (CQ) has seen its efficacy reduced in the late 60 s onwards after a decade of use. CQ resistance (CQR) was initially reported in Southeast Asia and South America, and then in Western Pacific and Africa^1^. The spread of CQR has led to successive antimalarial drug replacements and since 2000 to the progressive adoption of artemisinin-based combination therapies (ACTs). CQ remains highly effective to treat malaria caused by the other human-infecting *Plasmodium* parasite species^2^.

During the parasite intraerythrocytic development, hemoglobin from the host cytosol is internalized by the parasite and delivered into its digestive vacuole (DV), an acidic lysosome-like organelle. Degradation of hemoglobin in the DV generates toxic Fe^2+^-heme molecules that spontaneously oxidize to form membrane-disrupting Fe^3+^-heme^3^. To avoid this, the Fe^3+^-heme molecules are biocrystallized in the DV into non-toxic, chemically inert crystals known as hemozoin.

Quinoline-containing antimalarial drugs such as CQ, but also piperaquine (PPQ), amodiaquine and pyronaridine, are thought to primarily interfere with the conversion of toxic heme to non-toxic hemozoin crystal^4^. This process has been especially characterized for CQ, which is found in the DV at high concentrations^5–7^, leading to the accumulation of toxic Fe^2+^-heme and parasite death^8^.

The main genetic determinant of CQR is the *Plasmodium falciparum* chloroquine resistance transporter gene (*pfcrt*)^9,10^. *Pfcrt* encodes a 424 amino acid protein (PfCRT), consisting of 10 transmembrane helices (TMs)^10^, which is essential to the parasite intraerythrocytic development^11^. At least six independent origins of CQR mutations were reported^12^, with various mutant *pfcrt* haplotypes harboring different sets of 4 to 10 non-synonymous mutations such as the Asian Dd2 haplotype that later spread to Africa^1^. Mutant PfCRT molecules have acquired the ability to expel CQ out of the DV^13^. All CQR haplotypes carry the key K76T mutation that removes a positive charge in TM 1, suggesting a charge-dependent transport mechanism as CQ is di-protonated in the acidic DV^10,13^. The K76T mutation is always accompanied by additional mutations which may increase the CQR level and/or attenuate the fitness cost of resistance^14,15^. PfCRT remains a protein of utmost clinical importance since both ancient and novel PfCRT mutations are associated with altered *in vitro* antimalarial activity of some ACT drugs (PPQ, amodiaquine, lumefantrine, and artemisinin derivatives) and with ACT treatment failures^13,16–21^. For most of these drugs, the PfCRT-dependent pharmaco-modulation might operate through different mechanisms than for CQ^19,22,23^.

After two decades of research, the physiological role of PfCRT in the parasite biology is still questioned. Its secondary structure, location in the DV membrane and belonging to the Drug/Metabolite Transporter (DMT) superfamily support its participation in metabolite transport^10,24^. PfCRT was reported as a proton-coupled transporter recognizing some cationic substrates^25^, a Fe^2+^/Fe^3+^ transporter^26^, and playing a role in glutathione homeostasis^27,28^. Earlier studies have also reported PfCRT as a Cl^−^ channel mediator, a proton pump regulatory and an activator of Na^+^/H^+^ exchangers^29–31^.

Like DMT proteins^32^, PfCRT may presumably operate by a mechanism known as the alternate access model^33,34^. In such a model, the binding of substrate, maintained inside the transporter through a so-called occluded state, triggers a structural transition between two other conformations, the inward- and outward-facing states, thereby inducing the translocation of the substrate across the membrane. In the case of PfCRT, the binding site(s) of physiological substrate(s) and the different structural states of the transporter remain to be fully elucidated. Consistent with this, a PfCRT mutant structure was recently determined in an inward-facing state by cryo-electron microscopy (named here PfCRT^cryo-EM^, open-to-DV) and also predicted by modeling in an outward-facing state (PfCRT^OF^, open-to-cytoplasm; which were published during the revision process of this work)^35^.

Here, we hypothesized that combining structural and evolutionary information could help identify putative functional regions of PfCRT and also their link with drug resistance. We reasoned that codon or amino acid sites that are important for PfCRT function are especially conserved. First, we investigated the variability of the conservation level across the CRT-coding DNA sequence and phylogeny over *Plasmodium* evolution. To get a finer map of evolutionary constraints in a structural context, we also built models of the tertiary structure of PfCRT by homology modeling, based on high-resolution 3D structures of DMT proteins containing 10 TMs^32,36^. Two different PfCRT conformations were predicted: an inward-facing state (PfCRT^IF^ model; *i.e*. open-to-DV) and an occluded state (PfCRT^OC^ model; *i.e*. both vacuolar and cytoplasmic gates closed) in which an open cavity and a binding pocket were identified, respectively. Combined evolutionary and structural analyses identified several slowly evolving amino acid sites located in the open cavity (for PfCRT^IF^) and binding pocket (for PfCRT^OC^). Through a comparative structural approach with other experimentally-characterized DMTs, we further identified several amino acid sites of the binding pocket that may be involved in the physiological transport activity of PfCRT. Finally, we explored the location and structural/physicochemical impacts of CQR and PPQ resistance (PPQR) mutations through a series of *in silico* mutagenesis from the PfCRT^OC^ and PfCRT^IF^ models.

## Results

### CRT sequence dataset and phylogenetic tree analysis

To evaluate the selective regime acting on the coding-sequence and protein product of the *crt* gene, we first retrieved orthologous CRT sequences from public databases. Non-*Plasmodium* species have not been included in the study because we cannot confirm that PfCRT homologous protein(s) in those organisms are truly orthologous to PfCRT. As we had enough evolutionary information with CRTs from *Plasmodium*, we choose this smaller dataset to avoid introducing any putative bias in our evolutionary analyses^37^. Among the retrieved 24 *Plasmodium* CRT sequences, only that of the bird-infecting *P. relictum* appeared incomplete with an absence of twenty amino acid positions in the C-terminal region. The other sequences showed high conservation in length, ranging from 423 to 425 amino acids (Supplementary Table S1), resulting in the presence of few indels (8 amino acid positions) in the CRT multiple sequence alignment that were removed from subsequent analyses (Supplementary Fig. S1). The phylogenetic tree produced from the curated *crt* sequence alignment was highly congruent with the acknowledged phylogeny of *Plasmodium* species^38^, with four main monophyletic groups (bird parasites, primate parasites ‘*Plasmodium*’, rodent parasites and primate parasites ‘*Laverania*’) supported by high bootstrap values (≥90%; Supplementary Fig. S2).

### Variable levels of conservation across the *crt* gene sequence and phylogeny

The selective pressures acting on the CRT-coding DNA sequence were searched for by estimating the non-synonymous to synonymous substitution rate ratio *ω* (=*d*_N_/*d*_S_) across lineages (branch model) and codon sites (random-site models) using the *codeml* program (*ω* > 1 indicates positive selection whereas *ω* < 1 reflects purifying selection)^39^. The entire *crt* gene was found to evolve under a variable selective regime across *Plasmodium* phylogeny as the free-ratio model (that allows *ω* to vary along branches in the *crt* phylogenetic tree) had better fit to the data than the one-ratio null model M0 (which supposes only one *ω* value for all branches; *p* = 1.85 × 10^−32^; Table 1). Although purifying selection (*ω* < 1) was pervasive across the whole phylogeny, some specific branches showed evidence of positive selection (*ω* > 1 in *P. vinckei vinckei* or in the common ancestor of *P. fragile*, *P. knowlesi* and *P. coatneyi)*, suggesting few adaptive changes during *Plasmodium* evolution (Supplementary Fig. S3). The substitution rate ratio *ω* was also found to be heterogeneously distributed across *crt* codon sites as assessed by the M0:M3 comparison (*p* = 1.45 × 10^−133^; Table 1), but no specific codon site was detected to evolve under positive selection since the positive selection models M2a and M8 provided no better fits to the data than the neutral models M1a and M7, respectively (*p* = 1.00 in both cases; Table 1 and Supplementary Table S2). Under the best-fitted model M3, the sequence encoding the cytoplasmic N- and C-terminal extremities of CRT were found to be much less conserved than most of the remaining protein-coding DNA sequence (also noticeable by visual inspection of the CRT multiple sequence alignment provided in Supplementary Figure S1). Those extremities contained several sites that were found phosphorylated in PfCRT^40^, including T416 which notwithstanding appeared highly conserved. Altogether, the data indicate that much of the CRT protein has evolved under purifying selection over *Plasmodium* evolution.

**Table 1:** Results of PAML analyses.

Models	2Δℓ	df	*p* value	Conclusion
M0:free-ratio	260.20	44	1.85 × 10^−32^	*ω* heterogeneous among lineages of the phylogenetic tree
M0:M3	623.24	4	1.45 × 10^−133^	*ω* heterogeneous across codon sites
M1a:M2a	0.00	2	1.00	No codon sites detected to evolve under positive selection
M7:M8	0.00	2	1.00	No codon sites detected to evolve under positive selection
Partitions: cytoplasmic-half (n = 146 codon sites) *versus* vacuolar-half (n = 196 codon sites) sides				
A:B	14.59	1	1.33 × 10^−4^	Different nucleotide substitution rates among partitions
B:D	19.71	2	5.24 × 10^−5^	Heterogeneity of evolutionary parameters among partitions
C:E	18.87	2	7.97 × 10^−5^	Heterogeneity of evolutionary parameters among partitions
Partitions: non-TMs (n = 116 codon sites) *versus* TMs (n = 226 codon sites)				
A:B	58.32	1	2.22 × 10^−14^	Different nucleotide substitution rates among partitions
B:D	12.56	2	1.87 × 10^−3^	Heterogeneity of evolutionary parameters among partitions
C:E	10.77	2	4.58 × 10^−3^	Heterogeneity of evolutionary parameters among partitions

*ω*, non-synonymous (*d*_N_) to synonymous (*d*_S_) substitution rate ratio (*d*_N_/*d*_S_); 2∆ℓ, log-likelihood ratio of the two tested models; df, degrees of freedom; TMs, transmembrane helices; non-TMs, loops connecting two TMs. 2Δℓ was compared to a chi-squared table to determine the significance of the likelihood ratio tests. For the two series of partitioning tests shown here, codon sites were partitioned with the PfCRT^IF^ model and we discarded the poorly conserved N- and C-terminal regions, both located in the cytoplasm (this alteration was conservative). Similar results were obtained when codon sites were partitioned with the PfCRT^OC^ model (data not shown).

### Identification of DMT template structures to predict PfCRT tertiary structure by homology modeling

In order to get a finer map of evolutionary constraints in a structural context, we aimed to produce a tertiary structure of PfCRT by homology modeling. By reviewing the literature, we found that, since 2016, several high-resolution 3D structures of DMTs containing 10 TMs have been determined by X-ray diffraction in different conformational states: the nucleotide sugar GDP-mannose transporter Vrg4 resolved in an inward-facing state (PDB ID: 5oge)^36^, the triose-phosphate/phosphate translocator TPT resolved in an occluded state (in complex with 3-phosphoglycerate; PDB ID: 5y79)^32^, and the aromatic amino acids and exogenous toxic exporter YddG (PDB ID: 5i20)^41^. Phyre2^42^ and HHpred^43^ interactive servers identified these same three DMT structures as the current best structures to model PfCRT, with an e-value < 5.4 e^−21^ and a *confidence* criterion (which represents the probability that the match between the sequence and the template arises from a true relationship^42^) of 98.7% for the least acceptable of these three DMT structures (YddG; Supplementary Tables S3 and S4). Despite a low sequence identity between PfCRT and these transporters, reaching a maximum of 14% for the PfCRT-TPT comparison (Supplementary Table S5), homology modeling remains still feasible^44^ and the PfCRT tertiary structure may be predicted in both inward-facing and occluded states.

As an initial validity control, we evaluated the variability in sequence and structure across the three candidate DMT templates which structures were experimentally and therefore independently obtained. Of note, in the phylogeny of the DMT superfamily, they are located in different branches^45^. Accordingly, the paired sequence identities for these three template transporters were low, reaching for example only 13% between TPT and Vrg4 (Supplementary Table S5). However, a visual inspection of paired structural superposition revealed a very similar fold (Supplementary Fig. S4). To quantify this, we computed for paired DMT structures the Cɑ-based root-mean-square deviation (RMSD, which measures to what extent a given residue in a protein of interest changes its position compared to the structurally aligned residue from another protein). When the TMs-connecting loops were included, the RMSD ranged from 5.3 Ångström (Å) for Vrg4-YddG to 7.6 Å for TPT-YddG, and respectively diminished to 4.5 Å and 5.4 Å after exclusion of the loops (Supplementary Table S5). Hence, these results indicated that the structures have a similar fold but a different degree of intra/extracellular gate openness. We also noted that these RMSD values are consistent with those observed for other transporter superfamilies. For example, we retrieved from the Protein Data Bank three members of the Major Facilitator Superfamily (MFS), LacY (PDB ID: 2y5y)^46^, GlpT (PDB ID: 1pw4)^47^ and EmrD (PDB ID: 2gfp)^48^ which, despite exhibiting low paired sequence identity (~15%), shared a similar fold and arrangement of 12 TMs^48^, with Cɑ-based RMSDs including TMs-connecting loops ranging from 5.9 Å (LacY-GlpT) to 8.9 Å (GlpT-EmrD; Supplementary Fig. S5). Therefore, we assumed that PfCRT may share a similar fold to the aforementioned 10 TMs-containing DMT proteins, at least regarding the membrane spanning region.

### Predicting different conformational states of PfCRT tertiary structure by homology modeling

Having identified robust DMT template structures, we modeled wild-type (WT) PfCRT tertiary structure in two different conformational states: an inward-facing state (PfCRT^IF^) and an occluded state (PfCRT^OC^), using respectively Vrg4 and TPT as templates (Supplementary Table S6)^32,36^. We discarded the YddG template because it had a lower sequence identity with PfCRT than Vrg4 (both in inward-facing state; Supplementary Table S5)^32,41^. We also discarded the PfCRT positions 269–313 corresponding to the long vacuolar loop connecting TM 7 and TM 8 and the cytoplasmic N- and C-terminal extremities (positions 1–55 and 399–424, respectively) because of insufficient amino acid coverage in DMT templates.

In homology modeling, alignment of the target-template sequences (here PfCRT-Vrg4 and PfCRT-TPT) is a critical step. Since hydrophobicity, which reflects the transmembrane topology, is typically conserved in TMs during evolution, the hydropathy profile can contain similar global features even in very distantly related proteins^49^. Consequently, we first produced a superfamily-averaged hydropathy profile alignment with AlignMe^50^ using the CRT multiple sequence alignment and an alignment of different members of the DMT superfamily. This profile-profile (CRTs-DMTs) alignment pinpointed some conserved amino acid positions that should be aligned in each final target-template alignment (Supplementary Figs. S6 and S7). We then produced a target-template alignment using the AlignMe PST algorithm which is designed for distantly related proteins (*i.e*. with a sequence identity <15%)^50^, and manually optimized it in order to align the conserved amino acid positions identified from the profile-profile alignment (see the final alignments for PfCRT-Vrg4 and PfCRT-TPT in Supplementary Figs. S6 and S7 respectively).

After the refinement and minimization steps, we checked the quality of the two built PfCRT models using local stereochemistry (MolProbity). For comparison, we used as controls several high-resolution 3D structures: the two DMT template and the PfCRT^cryo-EM^ structure^35^. The two PfCRT models exhibited similar quality indices than these experimentally-determined control structures as suggested by Ramachandran plots (Table 2). General (ERRAT^51^, Prosa II^52^) and transmembrane-specialized (QMEANBrane^53^) 3D quality metrics also provided high confidence in structure conformation, energies and non-bonded interactions between atoms of the PfCRT^OC^ and PfCRT^IF^ models, when compared to the templates and other highly refined structures including PfCRT^cryo-EM^ from the Protein Data Bank (Table 2 and Supplementary Fig. S8). Finally, directional atomic contacts were evaluated to check the structures for correctness using the fine packing quality control implemented in the What IF server^54^. For both PfCRT models, the average Z-score for all contacts was close to 0, confirming the normality of the local environment of amino acids, similarly to Vrg4 and TPT template structures and PfCRT^cryo-EM^ (Table 2). We then found that the paired PfCRT^IF^-Vrg4 and PfCRT^OC^-TPT structures had a Cɑ-based RMSD of 5.2 Å and 3.0 Å, respectively (3.0 Å and 2.5 Å when TMs-connecting loops were excluded; Supplementary Table S5). These RMSD values were similar to those obtained with paired DMT template structures (Supplementary Table S5). In all, different quality metrics and structural comparisons indicated that the PfCRT^IF^ and PfCRT^OC^ models can be reliably used for further structural analyses.

**Table 2:** Quality metrics for PfCRT models and templates.

Protein^a^	ProSA II^b^	MolProbity^c^						ERRAT^d^ [0-100]	QMEAN Brane^e^ [0-1]	Fine packing quality control^f^
		Poor rotamers, n (%)	Favored rotamers, n (%)	Ramachandran outliers, n (%)	Ramachandran favored, n (%)	Bad bonds, n (%)	Bad angles, n (%)			
Vrg4	−1.76	21/268 (7.84)	218/268 (81.34)	2/298 (0.67)	278/298 (93.29)	0/2415 (0.00)	4/3278 (0.12)	92.32	0.80	0.47
TPT	−3.17	5/251 (1.99)	239/251 (95.22)	0/303 (0.00)	300/303 (99.01)	0/2423 (0.00)	0/3313 (0.00)	96.61	0.84	0.85
PfCRT^Cryo-EM^	−2.75	3/312 (0.96)	289/312 (92.63)	0/346 (0.00)	334/346 (96.53)	0/2876 (0.00)	0/3898 (0.00)	89.10	0.81	−0.60
PfCRT^IF^	−2.72	4/279 (1.43)	269/279 (96.42)	0/301 (0.00)	287/301 (95.35)	0/2550 (0.00)	2/3446 (0.06)	98.70	0.79	0.24
PfCRT^OC^	−2.11	0/272 (0.00)	270/272 (99.26)	0/294 (0.00)	287/294 (97.62)	4/2487 (0.16)	3/3366 (0.09)	99.01	0.81	0.43

^a^The experimentally-determined tertiary structures of two DMTs (Vrg4 and TPT) were used as templates for homology modeling of PfCRT structure.PfCRT^cryo-EM^ corresponds to a PfCRT mutant structure recently determined in an inward-facing state by cryo-electron microscopy. PfCRT^IF^ and PfCRT^OC^ correspond to PfCRT wild type models in inward-facing and occluded states, respectively. ^b^ProSA II generates an overall quality score compared with the ones from all experimentally determined protein chains available in the Protein Data Bank. ^c^MolProbity indicates the accuracy of a macromolecular structure model and diagnoses local problems by all-atom contact analysis, complemented by updated versions of covalent-geometry and torsion-angle criteria. ^d^ERRAT analyzes the statistics of non-bonded interactions between different atom types, then plots error values as a function of the position of a 9-residue sliding window, calculated by a comparison with statistics of non-bonded atom-atom interactions from highly refined structures. ^e^QMEANBrane is a structure quality assessment tool dedicated to membrane proteins. It determines a local quality score per residue (the mean per-residue quality score is shown), by combining statistical potentials trained on membrane protein structures with a per-residue weighting scheme. ^f^The fine packing quality control, implemented in the What IF server, checks the normality of the local environment of amino acids. Values are expressed as Z-scores: a value < −2 or >2 indicates a wrong model or an incorrect structure; a Z-score close to 0 represents a high quality structure.

**Figure 1:**
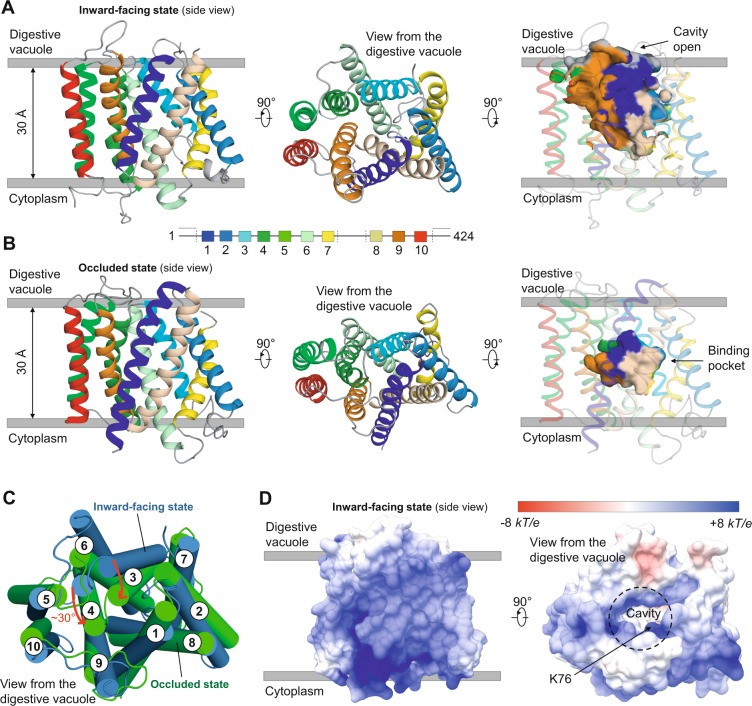
3D-fold and structural features of the wild type (WT) PfCRT^IF^ and PfCRT^OC^ models. Predicted tertiary structure of PfCRT^IF^ (**A**) and PfCRT^OC^ (**B**) models. Each TM is highlighted in a specific color. In PfCRT^IF^, the cavity is open from the vacuolar face. In PfCRT^OC^, a substrate-binding pocket constitutes the core of the transporter. (**C**) Superposition of PfCRT^IF^ and PfCRT^OC^ models. TMs are shown as cylinders. A prominent shift of ~30° (red arrows) was observed for both TM 3 and TM 4 between PfCRT^IF^ (blue) and PfCRT^OC^ (green). (**D**) Electrostatic surface potential of PfCRT^IF^. Values are in units of *kT*/e at 298 K, on a scale of −8 *kT*/*e* (red) to + 8 *kT*/*e* (blue). White color indicates a neutral potential. The structure is shown from the side (*left* structure) and the vacuolar face (*right* structure). The location of the key position K76, involved in CQR when mutated (K76T mutation), is indicated.

**Figure 2:**
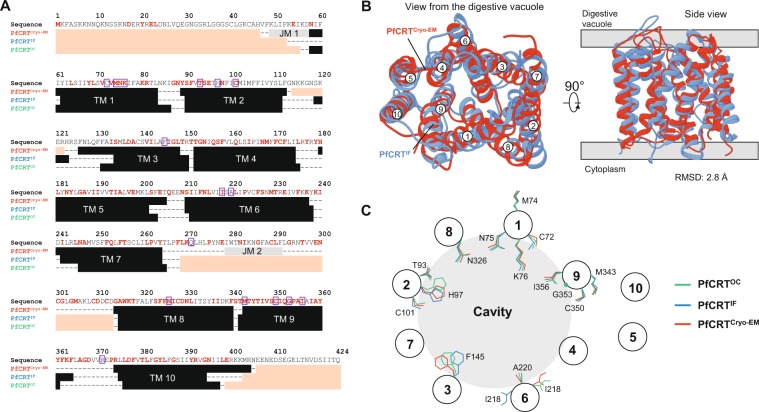
Structural comparison of PfCRT^cryo-EM^ with both PfCRT^IF^ and PfCRT^OC^ models. (**A**) Delineation of PfCRT secondary structures according to cryo-EM (PfCRT^cryo-EM^) or modeling (PfCRT^IF^ and PfCRT^OC^). TMs are colored in black, whilst TMs-connecting loops are indicated with dashes. Non-TM helices are shown in grey. Regions that are not covered by the PfCRT structures are colored in salmon. Strictly conserved CRT amino acid positions across *Plasmodium* species are written in red. Positions associated with CQR or PPQR mutations are surrounded in purple. (**B**) Structural superposition of PfCRT^cryo-EM^ with the PfCRT^IF^ model. For ease of representation, the long vacuolar loop connecting TM 7 and TM 8 was removed in PfCRT^cryo-EM^. The structures are shown as cartoon from the digestive vacuole (*left* structure) and the side (*right* structure). PfCRT^cryo-EM^ and PfCRT^IF^ are respectively colored in red and blue. The labels of TMs are also indicated. Both superposition and RMSD calculation were based on all Cɑ atoms (including TMs-connecting loops) using the *MatchMaker* function implemented in UCSF Chimera. When TMs-connecting loops were excluded, the RMSD was 2.4 Å. (**C**) Schematic representation of the side-chain orientation of the positions associated with CQR or PPQR mutations. The side-chain orientations were noted by visual inspection of a structural superposition of PfCRT^cryo-EM^, PfCRT^IF^ and PfCRT^OC^. Mutant positions in PfCRT^cryo-EM^ (S72, T76, S220, D326 and L356) were substituted to WT (C72, K76, A220, N326 and I356) using the *swapaa* function of UCSF Chimera. TM 5 and TM 10 are not involved in the architecture of the transporter cavity.

To evaluate the accuracy of the PfCRT^IF^ and PfCRT^OC^ models, we compared their secondary and tertiary structural features with those of PfCRT^cryo-EM^. Our two models revealed symmetry-related structural repeats consisting of 10 TM bundle (with TM 1 to TM 5 and TM 6 to TM 10 distributed in anticlockwise and clockwise manners, respectively), approximately 30 Å in length, similarly to PfCRT^cryo-EM^ (Fig. 1A,B). In PfCRT^cryo-EM^, two additional helices (JM 1 and JM 2) were identified, respectively located in the N-terminal region and the long vacuolar loop connecting TM 7 and TM 8 (two regions discarded in our modeling study; Fig. 2A). In the PfCRT^IF^ model, the structure was basket-shaped, similarly to Vrg4 and PfCRT^cryo-EM^, with a deep cavity of ~3,200 Å^3^ opened at the vacuolar side and composed of amino acids from most TM helices except TM 5 and TM 10 (Fig. 1A). In the PfCRT^OC^ model, a pocket of ~1,050 Å^3^ was observed in the core of the structure (Fig. 1B; the 41 pocket positions are listed in Supplementary Table S7), which corresponds to the substrate-binding pocket in TPT^32^. A structural comparison of PfCRT^IF^ and PfCRT^OC^ revealed a prominent difference in the orientation of TM 3 and TM 4 of ~30° at the vacuolar face (Fig. 1C), suggesting that these helices undergo rocker-switch movements to open and close the vacuolar gate. By estimating the electrostatic surface potential on the models (which have WT PfCRT haplotype), we noted that the vacuolar gate is slightly electropositive whilst the cavity and putative binding pocket are globally neutral (Fig. 1D). TMs boundaries between PfCRT^cryo-EM^ and models were similar, except TM 3, TM 7 and TM 10 that were longer in PfCRT^cryo-EM^ (Fig. 2A). In addition, the folds of the PfCRT^IF^ model and PfCRT^cryo-EM^ were highly similar as evidenced by a RMSD of 2.8 Å (2.4 Å without TMs-connecting loops; Fig. 2B), which provides a direct validation of our PfCRT^IF^ model. This result is consistent with the Cɑ-based structural conservation profile produced between PfCRT models and PfCRT^cryo-EM^ using the Dali server^55^, revealing a weaker structural conservation only on some TMs-connecting loops (Supplementary Fig. S9). Also, Cɑ-based contact maps generated with CMWeb^56^ indicated overall the same intra-protein contacts between our PfCRT models and PfCRT^cryo-EM^, with similar pairwise amino acid contact distributions (Supplementary Fig. S10). Finally, we used the orientation of side-chain amino acids related to CQR and PPQR mutations as another indicator of validity. After substituting mutant positions in PfCRT^cryo-EM^ (S72, T76, S220, D326 and L356) to WT (C72, K76, A220, N326 and I356) with UCSF Chimera^57^, we noticed that most of drug resistance-associated amino acid sites were similarly oriented in the PfCRT models compared to PfCRT^cryo-EM^ (Fig. 2C), except a major difference for the amino acid I218 in PfCRT^IF^. Altogether, the two models we produced were accurate and reliable for subsequent analyses.

### Vacuolar and TM sites have evolved under strong purifying selection

We used our new PfCRT models to test whether some PfCRT regions, apart from the N- and C-terminal extremities, were differentially conserved. We used the fixed-site models and partitioning function implemented in the *codeml* program^58^. We tested two different partitioning of *crt* codon sites: *1)* cytoplasmic-half *versus* vacuolar-half sides of the transporter and *2)* non-TMs (*i.e*. loops) *versus* TMs. In the two sets of partitioning, model B provided a better fit to the data than model A which assumes the same substitution pattern with identical parameters among partitions (*p* = 1 × 10^−4^ and 2 × 10^−14^ for cytoplasmic-half/vacuolar-half and non-TMs/TMs, respectively, Table 1 and Supplementary Table S8). This indicates that the nucleotide substitution rates at vacuolar and TM sites were on average 0.80 and 0.62 times as low as at the cytoplasmic and non-TM sites, respectively. More complex models D and E also provided better fits to the data than comparison models B and C (Table 1), confirming a heterogeneity of evolutionary parameters in the two sets of partitioning (Supplementary Table S8). By looking at the substitution rates *ω* in the different partitions, vacuolar and TM sites were under stronger purifying selection than cytoplasmic and non-TM sites, respectively (cytoplasmic-half/vacuolar-half: *ω*_1_ = 0.121/*ω*_2_ = 0.070, best fitted D model; non-TMs/TMs: *ω*_1_ = 0.111/*ω*_2_ = 0.078, best fitted E model; Supplementary Table S8). This is consistent with TMs being the hydrophobic and conserved core of the protein and the vacuolar side being under functional constraint as the likely entry route for physiological substrates^6,13,22,25,26,59^.

### The cavity of PfCRT contains highly conserved amino acid sites

**Figure 3:**
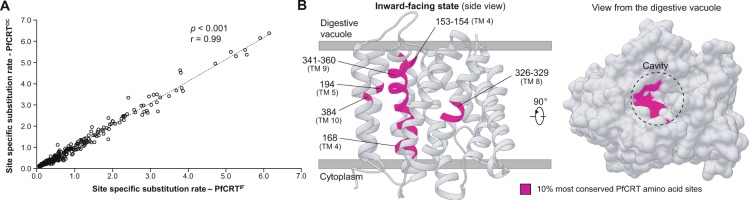
Location of the most conserved amino acid sites in the PfCRT tertiary structure. (**A**) Positive correlation of site-specific substitution rates estimated by the FuncPatch server between PfCRT^OC^ and PfCRT^IF^ models. Each point corresponds to one amino acid site. (**B**) Location of the 10% most conserved amino acid sites (inferred with FuncPatch) in the PfCRT^IF^ model, which are colored in dark pink. The structure is shown as cartoon from the side (*left* structure) and as surface from the digestive vacuole (*right* structure).

We next searched for the most conserved amino acid positions in the PfCRT^OC^ and PfCRT^IF^ models as indicators of putative functional sites. We used the FuncPatch server which jointly analyses phylogenetic tree, protein tertiary structure and conservation information. FuncPatch infers site-specific substitution rates at the protein level by taking into account their spatial location in the tertiary structure, and is especially useful in the case of highly conserved proteins^37,60^. Site-specific substitution rates were very similar using either the PfCRT^IF^ or PfCRT^OC^ models (Spearman’s rank correlation: r = 0.99, *p* < 0.001; Fig. 3A and Supplementary Data S1), so only the data obtained with PfCRT^IF^ will be presented. We detected a significant spatial correlation of site-specific substitution rates in the PfCRT^IF^ model as evidenced by a log Bayes factor of 47.6, suggesting the presence of a functional patch. We then mapped onto the tertiary structure the 10% most conserved amino acid sites of the 297 PfCRT positions included in the FuncPatch analysis. Remarkably, almost the whole TM 9 that lines the PfCRT^IF^ cavity was highlighted (Fig. 3B and Supplementary Table S9). Other highly conserved positions were located on TM 4, TM 5, in the middle of TM 8 and on TM 10 (Fig. 3B). It is noteworthy that TM 9 has been reported to play a major role in substrate binding in other DMTs consisting of 10 TMs^24^, and especially in Vrg4 and TPT proteins (Supplementary Fig. S11)^32,36^. Evolutionary rates at the protein level were also estimated using the Consurf web server^61^, and this analysis revealed again the high conservation of the PfCRT^IF^ cavity and especially TM 9 (Supplementary Fig. S12).

### PfCRT amino acids Y68, D329, Y345 and S349 might play a role in physiological substrate trafficking

**Figure 4:**
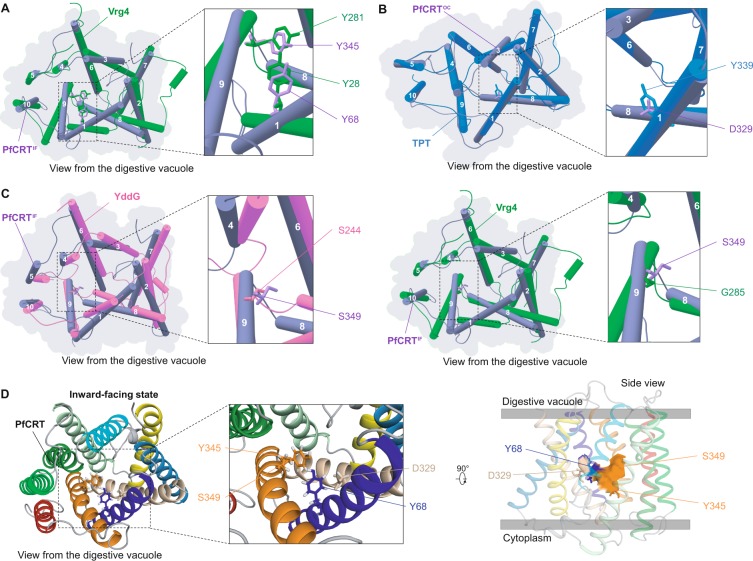
Structural alignment of PfCRT models with experimental DMT structures for the identification of putative binding sites in PfCRT pocket. Structural alignment of PfCRT^IF^ or PfCRT^OC^ (purple) with (**A** & **C**) Vrg4 (green forest), (**B**) TPT (blue) and (**C**) YddG (pink). TMs are numbered 1 to 10. PfCRT^IF^ was aligned with Vrg4 and YddG, while PfCRT^OC^ was aligned with TPT, using the *MatchMaker* function in UCSF Chimera. The different amino acid sites from Vrg4, TPT and YddG shown here were previously demonstrated to play a crucial role in substrate trafficking^32,36,41^. (**D**) Location of the four putative PfCRT functional sites Y68, D329, Y345 and S349 in PfCRT^IF^. The structure is shown as cartoon from the digestive vacuole (*left* panel) with a zoom on the proposed functional positions (*central* panel). Candidate functional positions are shown in sticks. The four amino acid positions cover a large part of the putative binding pocket in the PfCRT^OC^ model (*right* panel).

Some of the most highly conserved PfCRT amino acid sites might be under strong functional – rather than structural – constraints. Being a member of the DMT superfamily, PfCRT may bind a substrate through amino acids located in its central cavity, and more particularly in the binding pocket. Consequently, we focused on amino acid positions strictly conserved across *Plasmodium* species and whose side-chains line the binding pocket. After these filtering steps, we listed 24 amino acid positions (Supplementary Table S10) that were further analyzed in three paired structural alignments, PfCRT^IF^-Vrg4, PfCRT^IF^-YddG and PfCRT^OC^-TPT. We searched for structurally aligned positions that have been shown experimentally to modulate substrate trafficking in DMT template proteins (Supplementary Fig. S13)^32,36,41^. Two tyrosines of PfCRT^IF^ (Y68 in TM 1 and Y345 in TM 9) paired with two tyrosines of Vrg4 (Y28 in TM 1 and Y281 in TM 9; Fig. 4A) reported to be essential for the translocation of GDP–mannose and GMP molecules^36^. One aspartic acid of PfCRT^OC^ (D329 in TM 8) paired with a tyrosine of the TPT protein (Y339 in TM 8, Fig. 4B) which is crucial for phosphate recognition and transport^32^. Finally, PfCRT^IF^ S349 (TM 9) paired with both S244 from the bacterial YddG protein and G285 from Vrg4 (Fig. 4C), which play a role in the transport of threonine/methionine and both GDP-mannose and GMP molecules, respectively^36,41^. Of those four candidate PfCRT positions, three belonged to the conserved patch evidenced by the FuncPatch server (D329, Y345 and S349; Supplementary Table S9). In PfCRT^cryo-EM^ (inward-facing state), the side-chains of these four positions were also oriented towards the cavity (Supplementary Fig. S14). Overall, this comparative evolutionary and structural analysis identified amino acid sites putatively involved in the physiological transport activity of PfCRT (Fig. 4D).

### Most PfCRT mutations conferring drug resistance are located in the cavity or binding pocket

**Figure 5:**
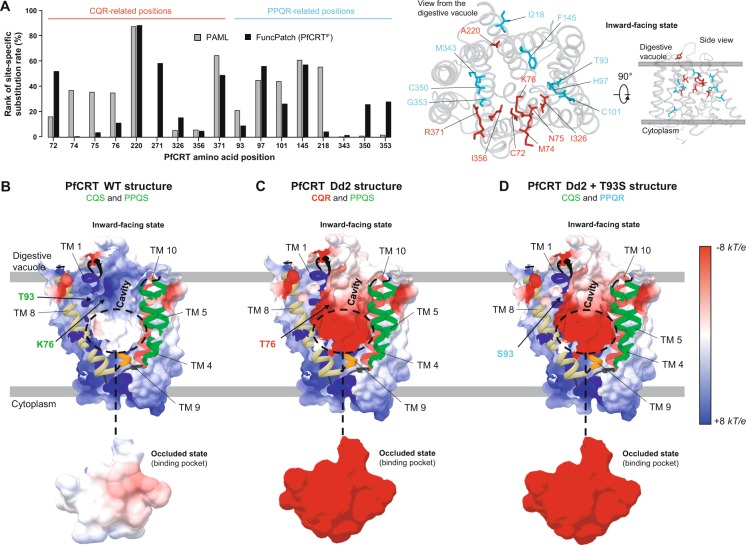
Location and physicochemical effects of drug resistance mutations on the PfCRT^OC^ and PfCRT^IF^ models. (**A**) *Left*: Conservation level of PfCRT amino acid positions associated with drug resistance. The conservation level of each position is expressed as the rank (in %) of its site-specific substitution rate estimated using either FuncPatch (with the PfCRT^IF^ model) or PAML. The lower the rank, the higher the conservation level. *Right*: Location of PfCRT amino acid positions associated with drug resistance in the PfCRT^IF^ model. CQR and PPQR mutations are shown in red and cyan, respectively. The electrostatic surface potential of the PfCRT cavity and binding pocket are shown for (**B**) WT, (**C**) CQR/PPQS and (**D**) CQS/PPQR structures. Values are in units of *kT*/e at 298 K, on a scale of −8 *kT*/*e* (red) to + 8 *kT*/*e* (blue). White color indicates a neutral potential. For each structure are indicated: the TMs (colored according to the Fig. 1A,B), the phenotype (CQR, chloroquine resistance; CQS, chloroquine sensitivity; PPQR, piperaquine resistance; PPQS, piperaquine sensitivity) and the mutations introduced in the tertiary structure (for ease of representation, only the K76T and T93S mutations are shown for the PfCRT^IF^ Dd2 and DD2 + T93S models). Only one viewing angle of the PfCRT cavity is shown here, but any other viewing angle exhibited the same electrostatic surface potential. Note that the Q271E mutation was not included in the mutant structures since it is located in the long TM 7-TM 8 vacuolar loop that was discarded from the PfCRT^OC^ and PfCRT^IF^ models.

Mutations in PfCRT confer drug resistance, chiefly to CQ and PPQ, but also alter sensitivities to other ACT drugs. To better understand the underlying resistance mechanisms, we mutated the PfCRT^OC^ and PfCRT^IF^ models *in silico*. We introduced jointly several mutations to produce Dd2, GB4 and 7G8 mutant structures which confer CQR and correspond to Asian, African and South American CQR haplotypes, respectively (Table 3). From an evolutionary view, five of the nine CQR-related positions investigated here were strictly conserved during *Plasmodium* evolution including the position 76 related to the key CQR K76T mutation (Supplementary Fig. S1). We noted that some positions related to CQR mutations (M74I, N75E and K76T) were markedly more conserved when site-specific substitution rates were computed by taking into account their spatial location in the tertiary structure (FuncPatch *versus* PAML data in Fig. 5A; Supplementary Data S1), indicating that these three amino acid sites were spatially close to conserved ones. Remarkably, the CQR-related positions we analyzed were directly located in the cavity of the PfCRT^IF^ model, except position 74 (mutation M74I) which lies in TM 1 but on the outer surface of the transporter and position 371 (mutation R371I) in the TM 9-TM 10 vacuolar loop next to the entry of the cavity (Figs. 2C and 5A). Based on the PfCRT^OC^ model, six positions related to CQR mutations were predicted to be involved in the architecture of the putative binding pocket (C72S, N75E, K76T, A220S, N326S/D and I356T/L), including five of the eight CQR mutations carried by the canonical Dd2 haplotype (Table 3). As the key CQR K76T mutation removes a positive charge in the cavity, we investigated *in silico* the changes in the electrostatic surface potential of the PfCRT^IF^ and PfCRT^OC^ Dd2, GB4 and 7G8 models. While the surface potential of PfCRT^IF^ WT cavity and PfCRT^OC^ WT binding pocket were neutral (Fig. 5B), they turned highly electronegative in all CQR mutant structures (Fig. 5C and Supplementary Fig. S15A).

**Table 3:** PfCRT haplotypes investigated in this study.

Origin region^a^	Parasite line^b^	Drug response^c^	TM 1	TM 1	TM 1	TM1	TM 2	TM 2	TM 2	TM 3	TM 6	TM 6	L7-8	TM 8	TM 9	TM 9	TM 9	TM 9	L9-10
			72	74	75	76	93	97	101	145	218	220	271	326	343	350	353	356	371
All regions	3D7	CQS, PPQS	C	M	N	K	T	H	C	F	I	A	Q	N	M	C	G	I	R
SE Asia	Dd2	CQR, PPQS	C	I	E	T	T	H	C	F	I	S	E	S	M	C	G	T	I
SE Asia	Dd2 + T93S	CQS, PPQR	C	I	E	T	S	H	C	F	I	S	E	S	M	C	G	T	I
SE Asia	Dd2 + H97Y	CQS, PPQR	C	I	E	T	T	Y	C	F	I	S	E	S	M	C	G	T	I
SE Asia	Dd2 + C101F	CQS, PPQR	C	I	E	T	T	H	F	F	I	S	E	S	M	C	G	T	I
SE Asia	Dd2 + F145I	CQS, PPQR	C	I	E	T	T	H	C	I	I	S	E	S	M	C	G	T	I
SE Asia	Dd2 + I218F	CQS, PPQR	C	I	E	T	T	H	C	F	F	S	E	S	M	C	G	T	I
SE Asia	Dd2 + M343L	CQS, PPQR	C	I	E	T	T	H	C	F	I	S	E	S	L	C	G	T	I
SE Asia	Dd2 + G353V	CQS, PPQR	C	I	E	T	T	H	C	F	I	S	E	S	M	C	V	T	I
S America	7G8	CQR, PPQS	S	M	N	T	T	H	C	F	I	S	Q	D	M	C	G	L	R
S America	7G8 + C350R	CQS, PPQR	S	M	N	T	T	H	C	F	I	S	Q	D	M	R	G	L	R
Africa	GB4	CQR, PPQS	C	I	E	T	T	H	C	F	I	S	E	N	M	C	G	I	I

^a^Geographic origin of the haplotype. SE Asia, Southeast Asia; S America, South America. ^b^For Dd2 and 7G8 parasite lines, one additional mutation was found to reverse CQR but confer PPQR^19,63^. ^c^CQS, chloroquine sensitivity; CQR, chloroquine resistance; PPQS, piperaquine sensitivity; PPQR, piperaquine resistance. In this study, the Q271E mutation was not introduced in the mutant structures and subsequent analyses since it is located in the long TM 7-TM 8 vacuolar loop that was discarded for tertiary structure prediction. The location in the structure (TM, transmembrane helix; L, loop) is indicated above each position.

Next, the recently identified, additive PfCRT mutations conferring PPQR on a CQR genetic background (namely T93S, H97Y, C101F, F145I, I218F, M343L and G353V, found in PfCRT Dd2; and C350R found in PfCRT 7G8)^19,62,63^ were individually introduced in the PfCRT^IF^ and PfCRT^OC^ Dd2 or 7G8 CQR mutant models. Similarly as CQR-related positions, PPQR-related positions were almost all located in the cavity of the PfCRT^IF^ model, except I218F and M343L which were located at the outer surface and at the cytoplasmic face of the transporter respectively (Figs. 2C and 5A). In the PfCRT^OC^ model, positions related to PPQR mutations H97Y, C101F, F145I, C350R and G353V were involved in the architecture of the binding pocket. We observed a large electronegative surface potential in the cavity and binding pocket, similarly to the PfCRT^IF^ and PfCRT^OC^ Dd2, GB4 or 7G8 mutant structures conferring CQR (Fig. 5D and Supplementary Fig. S15B). Of note, addition of C350R rendered neutral a large part of the cavity except in the proximity of K76T (note that this was found only in the PfCRT^IF^ model; Supplementary Fig. S15B). Six of the eight PPQR-related positions were strictly conserved during *Plasmodium* evolution (T93, C101, I218, M343, C350, G353; Supplementary Fig. S1).

Altogether, we concluded that PfCRT positions associated with mutations conferring resistance to quinoline-containing antimalarial drugs were globally conserved over *Plasmodium* evolution, mostly located in the cavity/binding pocket where physiological substrate trafficking is supposed to take place, and made the surface potential of the cavity and binding pocket more electronegative, as reported using the high-resolution PfCRT^cryo-EM^ structure^35^.

## Discussion

Based on our evolutionary results, we found that PfCRT is on average more conserved on its vacuolar-half side and its membrane-spanning domain. Therefore, the vacuolar side of PfCRT may play an important role in substrate recognition and/or transport, which supports the hypothesis of a physiological substrate being expelled by PfCRT out of the parasite DV. This is in line with the observation that recombinant PfCRT, either reconstituted in proteoliposomes^25^ or expressed at the surface of *Xenopus laevis* oocytes^13,26^, directionally transports CQ, peptides, basic amino acids, and ferric/ferrous iron. In the parasite, these solutes are known to accumulate in the DV from which they could be transported out either solely by mutant PfCRT (CQ and PPQ in their protonated forms) or by WT and/or mutant PfCRT (peptides, basic amino acids, and ferric/ferrous iron).

A high-resolution 3D PfCRT mutant structure determined in an inward-facing state by single-particle cryo-electron microscopy (PfCRT^cryo-EM^) was recently published^35^. The authors also modeled an outward-facing conformation of this mutant PfCRT (PfCRT^OF^), considering the pseudo-symmetrical arrangement of the TMs. Here, we complete the putative cycle of the PfCRT transporter by producing a model of PfCRT in an occluded state (PfCRT^OC^), where any substrate is engulfed by the residues of the binding pocket. Altogether, the results are compatible with the hypothesis that PfCRT may operate by the alternate access model^33,34^. Such a transporter mechanistic model was already proposed for other DMT proteins^32^. In PfCRT, the vacuolar half of TM 3 and TM 4 undergo rocker-switch movements which may contribute to open (in PfCRT^IF^/PfCRT^cryo-EM^) and close (PfCRT^OC^ and PfCRT^OF^) the vacuolar gate. Meanwhile, based on the PfCRT^OF^ model from Kim *et al*. (2019)^35^, the cytoplasmic half of TM 8 and TM 9 act similarly and may contribute to open (in PfCRT^OF^) and close (PfCRT^IF^/PfCRT^cryo-EM^ and PfCRT^OC^) the cytoplasmic gate.

Based on evolutionary conservation and structural alignment with other DMTs, we also propose four PfCRT candidate sites in the binding pocket that may be involved in the transport of physiological substrate(s): Y68 (TM 1), D329 (TM 8), Y345 (TM 9) and S349 (TM 9). None of these positions were found mutated in drug-resistant parasites. Mutations at those positions could be either too deleterious for the parasite physiology and survival (*pfcrt* is an essential gene^11^) or not involved in drug transport. Two other interesting sites in TM 8 (N326) and TM 9 (I356) from the highly conserved patch are located in the PfCRT binding pocket. N326 and I356 were initially mutated in the canonical Dd2 haplotype that initially invaded Southeast Asia and Africa. Their removal does not significantly alter CQR but enhances parasite fitness in *pfcrt*-modified isogenic parasite lines^20^. These N326/I356-revertant fitter haplotypes are now predominant in sub-Saharan Africa, suggesting some competitive advantage of carrying WT residues at those positions^20^.

The modeled PfCRT^IF^ and PfCRT^OC^ structures predicted here provide some insights into quinoline-containing drug resistance, which are fully congruent with those recently reported^35^. The most striking result is that most positions mutated in CQR, including the key K76T, are located in the cavity of the PfCRT^IF^ model, and more particularly in its predicted substrate-binding pocket (PfCRT^OC^ model). Several of the mutated positions (C72S, M74I, N75E, N326S/D, I356T/L) are in close proximity with K76T, suggesting that all these mutations work together. These amino acid changes jointly cause drastic alterations of the electrostatic surface potential in the vacuolar entry of the cavity and in the binding pocket that become highly electronegative in the CQR PfCRT mutant structures. This radical shift in electrostatic surface potential is very likely a major contributor to the dramatically increased uptake of di-protonated CQ in *X. laevis* oocytes expressing CQR *versus* WT PfCRT^13^ and the decreased accumulation of CQ in the DV of CQ-resistant parasites^6,64^.

Similarly to CQR mutations, most of the novel PPQR mutations that occurred in Dd2 or 7G8 CQR genetic backgrounds^19,21,62,63^ are located in the cavity and binding pocket in our PfCRT^IF^ and PfCRT^OC^ models. However, the addition of PPQR mutations on CQR mutant structures (Dd2 and 7G8 haplotypes) did not alter the electronegative surface potential. This was moderately surprising because some PPQR mutations (such as M343L or G353V) attenuate but do not fully reverse CQR^19^. Remarkably, substantially increased PPQ transport rates by PPQ-resistant PfCRT variants were found in proteoliposomes, which were then confirmed in *pfcrt*-edited parasites^35^. Hence, we hypothesize that PPQ-conferring mutations provide additional changes in the cavity/binding pocket which could affect interactions with PPQ.

In conclusion, we showed that our bioinformatics analysis of PfCRT led to results similar to those obtained with the recently obtained experimental structure^35^ regarding the overall fold, architecture of the open cavity and alteration of its electrostatic surface potential in drug-resistant PfCRT mutant structures. Such results strengthen the usefulness of tertiary structure prediction of transmembrane proteins through homology modeling, even when a protein to model shares less than 15% sequence identity than a similarly folded template structure. Furthermore, we complement the experimental work of Kim *et al*. (2019)^35^ by providing an occluded state model of PfCRT and identifying a putative binding pocket where a substrate is likely maintained and could trigger a structural transition between the inward- and outward-facing states. This pocket, of smaller volume than the open cavity, also provides with a shortened list of highly conserved putative functional sites to be experimentally tested.

## Materials and Methods

### Collection of PfCRT orthologous sequences

The PfCRT amino acid sequence (PlasmoDB database identifier: PF3D7_0709000) was queried against the specialized *Plasmodium* database (PlasmoDB, release 38) and the non-redundant protein database of NCBI using blastp and tblastn searches (e-value cutoff = 10^−6^, BLOSUM62 scoring matrix)^65^. Twenty four CRT protein and corresponding cDNA sequences from distinct *Plasmodium* species were retrieved (Supplementary Table S1). For *P. ovale* spp. and *P. inui* sequences, exon skipping was manually performed so as to reconstruct the full cDNA and amino acid sequences.

### Multiple protein and codon sequence alignments

The CRT multiple sequence alignment was first generated with TM-aligner^66^ using the transmembrane-specific substitution matrix PHAT^67^ and with gap opening and extension penalties set to 10 and 1, respectively. The output alignment was visually inspected and manually edited using BioEdit v7.2.5^68^. The positions of the CRT multiple alignment containing gaps in ≥30% of all sequences were removed. Then, a *crt* nucleotide sequence alignment was generated with PAL2NAL^69^ using the cleaned CRT protein sequence alignment as template.

### Phylogenetic analysis

A phylogenetic tree was built from the *crt* nucleotide sequence alignment by using the maximum likelihood method implemented in PhyML v3.0^70^, after determining the best-fitting nucleotide substitution model using the Smart Model Selection (SMS) package^71^. A general time-reversible model with optimized equilibrium frequencies, gamma distributed among-site rate variation and estimated proportion of invariant sites (GTR + *G* + *I*) was used, as selected by the Akaike Information Criterion. The nearest neighbor interchange approach implemented in PhyML was chosen for tree improving, and branch supports were estimated using the approximate likelihood ratio aLRT SH-like method^72^. The *crt* phylogenetic tree was rooted using the most distant bird-infecting *Plasmodium* species as outgroup and displayed using the iTOL server^73^.

### Analysis of selective pressures acting on ***crt***

To investigate the evolutionary regime that has shaped the CRT-coding DNA sequence during *Plasmodium* evolution, the nucleotide sequence alignment and the maximum likelihood phylogenetic tree were submitted to the *codeml* tool of PAML v.4.9h^39,74^. The level of coding sequence conservation was measured by calculating the non-synonymous (*d*_N_) to synonymous (*d*_S_) substitution rate ratio *ω* (=*d*_N_*/d*_S_). Theoretically, positive selection at codon sites is indicated by *ω* > 1, while codon sites associated with *ω* = 1 and *ω* < 1 suggest neutral evolution and purifying selection, respectively. We investigated the heterogeneity of *ω* among lineages and along the *crt* sequence; then we searched for codons evolving under positive selection using a set of random-site and branch models (free-ratio, M0, M1a, M2a, M3, M7 and M8)^75,76^. Model M0 supposes the same *d*_N_/*d*_S_ ratio for all branches of the phylogeny, while the “free-ratio” branch model assumes independent *d*_N_/*d*_S_ ratio for every branch. Model M1a allows two classes of sites under neutral evolution (*ω* = 1) and purifying selection (0 ≤ *ω* < 1), whereas model M2a adds a third class of sites to model M1a as positive selection (*ω* > 1). In the discrete model M3, several classes (*k* = [3–5] in this study) of independent *ω* were estimated, each of them being associated with a specific proportion. Model M7 assumes a β-distribution of ten *ω* ratios limited to the interval [0, 1] with two shape parameters *p* and *q*. In model M8, an additional site class is estimated, with *ω* possibly > 1 as M2a does. The heterogeneity of *ω* among codon sites was tested by comparing model M0 to model M3, while paired models M1a:M2a and M7:M8 allow to detect positively selected sites^75^. Candidate sites for positive selection were identified in M2a and M8 models using the Bayes empirical Bayes inference (BEB)^77^, which calculates the posterior probability that every codon site belongs to a site class affected by positive selection; and using the naïve empirical Bayes (NEB) inference in model M3 (in which no BEB approach is implemented yet), considered as less powered than the BEB inference. For models considering *k* classes of *d*_N_/*d*_S_ ratio, we used the mean *ω* value at each codon site, calculated as the sum of the *ω* values of each *k* class weighted by their estimated probabilities (Supplementary Data S1)^39^.

Statistical analyses based on structural information of the PfCRT^IF^ and PfCRT^OC^ models were performed using the partitioning function implemented in *codeml*^58^ in order to test *i*) whether the vacuolar-half side is more or less conserved than the cytoplasmic-half side; and *ii*) whether TMs are more or less conserved than non-TMs (or loops). Partitionings are based on fixed-site models A to E. Model A – that corresponds to model M0 – assumes identical parameters for transition/transversion (κ) rate ratio, branch lengths and equilibrium nucleotide frequencies, and unique *ω* ratio for partitions. Model B allows homogeneity among partitions in codon frequencies, κ and *ω* ratios but different rates of evolution, whereas model C further accounts for potential variation in codon frequency. Model D uses different κ and *ω* ratios based on the same codon frequencies for partitions, and model E ultimately allows different κ, *ω* and codon frequencies between partitions. The A:B comparison tests for different nucleotide substitution rates among partitions, while both B:D and C:E comparisons evaluate the variation in κ, *ω*, and codon frequencies among partitions^58^.

The comparisons of models were performed using Likelihood Ratio Tests (LRTs)^78^. For every LRT, twice the log-likelihood difference between alternative and null models (2Δℓ) was compared to critical values from a chi-squared distribution with degrees of freedom equal to the difference in the number of estimated parameters between both models^79^.

### Modeling the PfCRT tertiary structure

When the analyses were performed, no experimental structure was determined for PfCRT. Consequently, we aimed at modeling it in different conformational states using a template-based protein structure modeling approach. The PfCRT sequence was submitted to interactive Phyre2^42^ and HHpred^43^ servers to find the best available, appropriate templates. The nucleotide sugar GDP-mannose transporter Vrg4 (inward-facing state, PDB ID: 5oge)^36^ and the triose-phosphate/phosphate translocator TPT (occluded state, PDB ID: 5y79)^32^ were identified as sharing putative similar structural folds with PfCRT with confidence >99%. The very low sequence identity of these proteins with PfCRT, reaching only 14% with TPT, makes homology modeling challenging but still feasible^44^. In homology modeling, the sequence alignment of the target (here PfCRT) and the template (here other DMT proteins) is a critical step. Because hydrophobicity reflects the transmembrane topology and is typically conserved in TMs during evolution, we first produced a superfamily-averaged hydropathy profile alignment with AlignMe^50^ using the CRT multiple sequence alignment and an alignment of different members of the DMT superfamily. The profile (CRT)-profile (DMT) alignment pinpointed some amino acids which are supposed to be important to align in the final target-template sequence alignment (Supplementary Figs. S6 and S7). Finally, the target-template alignment was produced using the AlignMe PST algorithm, designed for distantly related proteins (*i.e*. with a sequence identity <15%)^50^, then manually optimized it in order to align the putative important amino acid positions identified from the superfamily-averaged hydropathy profile alignment (Supplementary Figs. S6 and S7). The long TM 7-TM 8 vacuolar loop and the cytoplasmic N- and C-terminal extremities were not considered for PfCRT modeling because of too few numbers of amino acid positions available from templates to properly cover them. Initially, we built 1,000 3D models for each PfCRT state satisfying the spatial restrains of the template structures using MODELLER 9.17^80^. The best models among them were selected based on scores calculated from discrete optimized protein energy (DOPE) and GA341 functions. Then, the best PfCRT^OC^ and PfCRT^IF^ models were submitted to GalaxyRefine^81^ for atomic-level, high-resolution refinement, which helped to achieve significant improvement in physical quality of the local structure. Finally, the refined models were subjected to an energy minimization to get the most stable conformations using the YASARA server^82^.

The refined, energy-minimized PfCRT models were then validated using *i*) MolProbity^83^ for local stereochemistry; *ii*) fine packing quality control implemented in the What IF server^54^ by estimating directional atomic contacts; and *iii*) ERRAT^51^, ProSA II^52^ and QMEANBrane^53^ for tertiary fold and global 3D quality metrics. Cɑ-based intra-protein contacts and pairwise amino acid contact distributions were investigated with the CMWeb server (default parameters)^56^. Finally, the Dali server^55^ was used to study the Cɑ-based structural conservation between the PfCRT models and the high-resolution PfCRT^cryo-EM^ structure. The PDB files of the refined, energy-minimized PfCRT^OC^ and PfCRT^IF^ models are provided in Supplementary Files S1 and S2, respectively.

### Analyses of structural features

Protein electrostatic surface potential was calculated using Adaptive Poisson-Boltzmann Solver (APBS)^84^, after determining the per-atom charge and radius of the structure with PDB2PQR v.2.1.1^85^. The Poisson-Boltzmann equation was solved at 298 K using a grid-based method, with solute and solvent dielectric constants fixed at 2 and 78.5, respectively. We used a scale of −8 *kT*/*e* to + 8 *kT*/*e* to map the electrostatic surface potential in a radius of 1.4 Å.

The cavity/pocket volume of the PfCRT models was analytically calculated using the Connolly’s surface (or molecular surface model) with CASTp 3.0 (default parameters)^86^.

Putative functional patches (*i.e*. highly conserved amino acid positions that are in close physical proximity in the tertiary structure) in the two PfCRT models were searched for using the FuncPatch server by estimating site-specific substitution rates, possibly spatially correlated^37^. We submitted to the server the PfCRT^OC^ and PfCRT^IF^ models along with the CRT multiple sequence alignment and the maximum-likelihood *crt* phylogenetic tree. The spatial correlation of the site-specific amino acid substitution rates in the PfCRT predicted structures was tested using a Bayesian model comparison, where a null model (model 0), in which no spatial correlation of site-specific substitution rates is present, is compared to the alternative model (model 1). As suggested by the FuncPatch’ authors, a spatial correlation was considered as significant if the estimated log Bayes factor (model 1 *versus* model 0) was larger than the conservative cutoff value of 8^37^. Finally, evolutionary rates were also estimated at each amino acid site of the PfCRT sequence and then mapped onto the tertiary structure with Consurf (default parameters)^61^, a widely used web server implementing advanced probabilistic evolutionary models. Contrary to FuncPatch, Consurf does not include the spatial correlation of site-specific substitution rates attributed to tertiary structure^37,61^.

### Identifying candidate functional sites of PfCRT

In order to identify candidate functional amino acid sites possibly involved in PfCRT physiological substrate(s) trafficking, a comparative evolutionary and structural analysis was performed to filter step-by-step PfCRT amino acid sites. Because functional amino acids are usually very conserved over evolutionary time^87^, only the strictly conserved amino acid positions (*i.e*. the absence of non-synonymous substitutions for a given position) of PfCRT during *Plasmodium* evolution were kept. From this subset of positions, we then focused on those involved in the putative substrate-binding pocket in the PfCRT^OC^ model since it is widely accepted that DMT proteins transport substrates through binding sites located in their pocket^24,32,36,41^. Finally, the DMT template structures used to predict the conformational states of PfCRT tertiary structure (Vrg4 and TPT)^32,36^, in addition with another DMT protein (YddG)^41^ were aligned with the PfCRT^OC^ and PfCRT^IF^ models. By focusing on the amino acid sites from these three structures that have been shown to be important or essential for substrate binding or transport, we listed PfCRT candidate positions that might contribute to substrate trafficking (Supplementary Fig. S13).

### *In silico* production of PfCRT mutant structures

Some *in silico* mutagenesis from the refined, energy-minimized PfCRT models were performed. We generated three PfCRT mutant models per PfCRT conformational state based on the Dd2, GB4 and 7G8 haplotypes which are associated with CQR (Table 3)^10,88^. The CQ-resistant PfCRT^IF^ or PfCRT^OC^ Dd2 and 7G8 structures were then subjected to individual additional mutations that attenuate CQR but confer PPQR (Table 3)^19,21,62,63^. Mutations were introduced using the *swapaa* function of UCSF Chimera by substituting the residue with the most probable rotameric conformation^57^.

### Structure visualization

Molecular drawings were produced using UCSF Chimera^57^.

## Supplementary information

Supplementary Figures and Tables.

Supplementary Files.

Supplementary Data S1.

## References

[1] Wootton JC (2002). Genetic diversity and chloroquine selective sweeps in Plasmodium falciparum. Nature.

[2] WHO | World malaria report *WHO*, http://www.who.int/malaria/publications/world-malaria-report-2018/report/en/ (2018).

[3] Milani KJ, Schneider TG, Taraschi TF (2015). Defining the morphology and mechanism of the hemoglobin transport pathway in Plasmodium falciparum-infected erythrocytes. Eukaryotic Cell.

[4] Kapishnikov S (2019). Mode of action of quinoline antimalarial drugs in red blood cells infected by Plasmodium falciparum revealed *in vivo*. Proc. Natl. Acad. Sci. USA.

[5] Yayon A, Cabantchik ZI, Ginsburg H (1984). Identification of the acidic compartment of Plasmodium falciparum-infected human erythrocytes as the target of the antimalarial drug chloroquine. EMBO J..

[6] Krogstad DJ, Gluzman IY, Herwaldt BL, Schlesinger PH, Wellems TE (1992). Energy dependence of chloroquine accumulation and chloroquine efflux in Plasmodium falciparum. Biochem. Pharmacol..

[7] Geng Y, Kohli L, Klocke BJ, Roth KA (2010). Chloroquine-induced autophagic vacuole accumulation and cell death in glioma cells is p53 independent. Neuro-oncology.

[8] Goldberg DE (1993). Hemoglobin degradation in Plasmodium-infected red blood cells. Semin. Cell Biol..

[9] Su X, Kirkman LA, Fujioka H, Wellems TE (1997). Complex polymorphisms in an approximately 330 kDa protein are linked to chloroquine-resistant P. falciparum in Southeast Asia and Africa. Cell.

[10] Fidock DA (2000). Mutations in the P. falciparum digestive vacuole transmembrane protein PfCRT and evidence for their role in chloroquine resistance. Mol. Cell.

[11] Zhang, M. *et al*. Uncovering the essential genes of the human malaria parasite Plasmodium falciparum by saturation mutagenesis. *Science* **360** (2018). 10.1126/science.aap7847 PMC6360947 29724925

[12] Wellems TE, Hayton K, Fairhurst RM (2009). The impact of malaria parasitism: from corpuscles to communities. J. Clin. Invest..

[13] Martin RE (2009). Chloroquine transport via the malaria parasite’s chloroquine resistance transporter. Science.

[14] Gabryszewski SJ (2016). Evolution of Fitness Cost-Neutral Mutant PfCRT Conferring P. falciparum 4-Aminoquinoline Drug Resistance Is Accompanied by Altered Parasite Metabolism and Digestive Vacuole Physiology. PLOS Pathogens.

[15] Lewis IA (2014). Metabolic QTL analysis links chloroquine resistance in Plasmodium falciparum to impaired hemoglobin catabolism. PLoS Genet..

[16] Sidhu ABS, Verdier-Pinard D, Fidock DA (2002). Chloroquine resistance in Plasmodium falciparum malaria parasites conferred by pfcrt mutations. Science.

[17] Cooper RA (2002). Alternative mutations at position 76 of the vacuolar transmembrane protein PfCRT are associated with chloroquine resistance and unique stereospecific quinine and quinidine responses in Plasmodium falciparum. Mol. Pharmacol..

[18] Valderramos SG (2010). Identification of a mutant PfCRT-mediated chloroquine tolerance phenotype in Plasmodium falciparum. PLoS Pathog..

[19] Ross LS (2018). Emerging Southeast Asian PfCRT mutations confer Plasmodium falciparum resistance to the first-line antimalarial piperaquine. Nature Communications.

[20] Dhingra SK (2019). Global Spread of Mutant PfCRT and Its Pleiotropic Impact on Plasmodium falciparum Multidrug Resistance and Fitness. mBio.

[21] van der Pluijm Rob W, Imwong Mallika, Chau Nguyen Hoang, Hoa Nhu Thi, Thuy-Nhien Nguyen Thanh, Thanh Ngo Viet, Jittamala Podjanee, Hanboonkunupakarn Borimas, Chutasmit Kitipumi, Saelow Chalermpon, Runjarern Ratchadaporn, Kaewmok Weerayuth, Tripura Rupam, Peto Thomas J, Yok Sovann, Suon Seila, Sreng Sokunthea, Mao Sivanna, Oun Savuth, Yen Sovannary, Amaratunga Chanaki, Lek Dysoley, Huy Rekol, Dhorda Mehul, Chotivanich Kesinee, Ashley Elizabeth A, Mukaka Mavuto, Waithira Naomi, Cheah Phaik Yeong, Maude Richard J, Amato Roberto, Pearson Richard D, Gonçalves Sónia, Jacob Christopher G, Hamilton William L, Fairhurst Rick M, Tarning Joel, Winterberg Markus, Kwiatkowski Dominic P, Pukrittayakamee Sasithon, Hien Tran Tinh, Day Nicholas PJ, Miotto Olivo, White Nicholas J, Dondorp Arjen M (2019). Determinants of dihydroartemisinin-piperaquine treatment failure in Plasmodium falciparum malaria in Cambodia, Thailand, and Vietnam: a prospective clinical, pharmacological, and genetic study. The Lancet Infectious Diseases.

[22] Bellanca S (2014). Multiple drugs compete for transport via the Plasmodium falciparum chloroquine resistance transporter at distinct but interdependent sites. J. Biol. Chem..

[23] Richards SN (2016). Molecular Mechanisms for Drug Hypersensitivity Induced by the Malaria Parasite’s Chloroquine Resistance Transporter. PLoS Pathog..

[24] Martin RE, Kirk K (2004). The malaria parasite’s chloroquine resistance transporter is a member of the drug/metabolite transporter superfamily. Mol. Biol. Evol..

[25] Juge N (2015). Plasmodium falciparum chloroquine resistance transporter is a H+-coupled polyspecific nutrient and drug exporter. Proc Natl Acad Sci USA.

[26] Bakouh N (2017). Iron is a substrate of the Plasmodium falciparum chloroquine resistance transporter PfCRT in Xenopus oocytes. J. Biol. Chem..

[27] Maughan SC (2010). Plant homologs of the Plasmodium falciparum chloroquine-resistance transporter, PfCRT, are required for glutathione homeostasis and stress responses. Proc. Natl. Acad. Sci. USA.

[28] Patzewitz E-M (2013). Glutathione transport: a new role for PfCRT in chloroquine resistance. Antioxid. Redox Signal..

[29] Zhang H, Howard EM, Roepe PD (2002). Analysis of the antimalarial drug resistance protein Pfcrt expressed in yeast. J. Biol. Chem..

[30] Zhang H, Paguio M, Roepe PD (2004). The antimalarial drug resistance protein Plasmodium falciparum chloroquine resistance transporter binds chloroquine. Biochemistry.

[31] Nessler S (2004). Evidence for activation of endogenous transporters in Xenopus laevis oocytes expressing the Plasmodium falciparum chloroquine resistance transporter, PfCRT. J. Biol. Chem..

[32] Lee Y (2017). Structure of the triose-phosphate/phosphate translocator reveals the basis of substrate specificity. Nat Plants.

[33] Mitchell P (1957). A General Theory of Membrane Transport From Studies of Bacteria. Nature.

[34] Jardetzky O (1966). Simple Allosteric Model for Membrane Pumps. Nature.

[35] Kim Jonathan, Tan Yong Zi, Wicht Kathryn J., Erramilli Satchal K., Dhingra Satish K., Okombo John, Vendome Jeremie, Hagenah Laura M., Giacometti Sabrina I., Warren Audrey L., Nosol Kamil, Roepe Paul D., Potter Clinton S., Carragher Bridget, Kossiakoff Anthony A., Quick Matthias, Fidock David A., Mancia Filippo (2019). Structure and drug resistance of the Plasmodium falciparum transporter PfCRT. Nature.

[36] Parker JL, Newstead S (2017). Structural basis of nucleotide sugar transport across the Golgi membrane. Nature.

[37] Huang Y-F, Golding GB (2015). FuncPatch: a web server for the fast Bayesian inference of conserved functional patches in protein 3D structures. Bioinformatics.

[38] Carlton JM (2018). Evolution of human malaria. Nat Microbiol.

[39] Yang Z (2007). PAML 4: phylogenetic analysis by maximum likelihood. Mol. Biol. Evol..

[40] Kuhn Y (2010). Trafficking of the phosphoprotein PfCRT to the digestive vacuolar membrane in Plasmodium falciparum. Traffic.

[41] Tsuchiya H (2016). Structural basis for amino acid export by DMT superfamily transporter YddG. Nature.

[42] Kelley LA, Mezulis S, Yates CM, Wass MN, Sternberg MJE (2015). The Phyre2 web portal for protein modeling, prediction and analysis. Nat Protoc.

[43] Söding J, Biegert A, Lupas AN (2005). The HHpred interactive server for protein homology detection and structure prediction. Nucleic Acids Res.

[44] Nikolaev DM (2018). A Comparative Study of Modern Homology Modeling Algorithms for Rhodopsin Structure Prediction. ACS Omega.

[45] Yen MR, Chen JS, Marquez JL, Sun EI, Saier MH (2010). Multidrug resistance: phylogenetic characterization of superfamilies of secondary carriers that include drug exporters. Methods Mol. Biol..

[46] Chaptal V (2011). Crystal structure of lactose permease in complex with an affinity inactivator yields unique insight into sugar recognition. Proc Natl Acad Sci USA.

[47] Huang Y, Lemieux MJ, Song J, Auer M, Wang D-N (2003). Structure and mechanism of the glycerol-3-phosphate transporter from Escherichia coli. Science.

[48] Yin Y, He X, Szewczyk P, Nguyen T, Chang G (2006). Structure of the Multidrug Transporter EmrD from Escherichia coli. Science.

[49] Lolkema JS, Slotboom D-J (1998). Estimation of structural similarity of membrane proteins by hydropathy profile alignment. Molecular Membrane Biology.

[50] Stamm M, Staritzbichler R, Khafizov K, Forrest LR (2014). AlignMe–a membrane protein sequence alignment web server. Nucleic Acids Res..

[51] Colovos C, Yeates TO (1993). Verification of protein structures: patterns of nonbonded atomic interactions. Protein Sci..

[52] Wiederstein M, Sippl MJ (2007). ProSA-web: interactive web service for the recognition of errors in three-dimensional structures of proteins. Nucleic Acids Res..

[53] Studer G, Biasini M, Schwede T (2014). Assessing the local structural quality of transmembrane protein models using statistical potentials (QMEANBrane). Bioinformatics.

[54] Vriend G (1990). WHAT IF: A molecular modeling and drug design program. Journal of Molecular Graphics.

[55] Holm L, Rosenström P (2010). Dali server: conservation mapping in 3D. Nucleic Acids Res..

[56] Kozma D, Simon I, Tusnády GE (2012). CMWeb: an interactive on-line tool for analysing residue-residue contacts and contact prediction methods. Nucleic Acids Res..

[57] Pettersen EF (2004). UCSF Chimera—A visualization system for exploratory research and analysis. J. Comput. Chem..

[58] Yang Z, Swanson WJ (2002). Codon-substitution models to detect adaptive evolution that account for heterogeneous selective pressures among site classes. Mol. Biol. Evol..

[59] Lee AH (2018). Evidence for Regulation of Hemoglobin Metabolism and Intracellular Ionic Flux by the Plasmodium falciparum Chloroquine Resistance Transporter. Sci Rep.

[60] Coppée R, Jeffares DC, Miteva MA, Sabbagh A, Clain J (2019). Comparative structural and evolutionary analyses predict functional sites in the artemisinin resistance malaria protein K13. Sci Rep.

[61] Ashkenazy H (2016). ConSurf 2016: an improved methodology to estimate and visualize evolutionary conservation in macromolecules. Nucleic Acids Res..

[62] Dhingra, S. K. *et al*. A Variant PfCRT Isoform Can Contribute to Plasmodium falciparum Resistance to the First-Line Partner Drug Piperaquine. *MBio* **8** (2017). 10.1128/mBio.00303-17 PMC5424201 28487425

[63] Pelleau S (2015). Adaptive evolution of malaria parasites in French Guiana: Reversal of chloroquine resistance by acquisition of a mutation in pfcrt. Proc. Natl. Acad. Sci. USA.

[64] Sanchez CP, Stein W, Lanzer M (2003). Trans stimulation provides evidence for a drug efflux carrier as the mechanism of chloroquine resistance in Plasmodium falciparum. Biochemistry.

[65] Altschul SF, Gish W, Miller W, Myers EW, Lipman DJ (1990). Basic local alignment search tool. J. Mol. Biol..

[66] Bhat B, Ganai NA, Andrabi SM, Shah RA, Singh A (2017). TM-Aligner: Multiple sequence alignment tool for transmembrane proteins with reduced time and improved accuracy. Sci Rep.

[67] Ng PC, Henikoff JG, Henikoff S (2000). PHAT: a transmembrane-specific substitution matrix. Predicted hydrophobic and transmembrane. Bioinformatics.

[68] Hall T (1999). BioEdit: a user-friendly biological sequence alignment editor and analysis program for Windows 95/98/NT. Nucleic Acids Symposium Series.

[69] Suyama M, Torrents D, Bork P (2006). PAL2NAL: robust conversion of protein sequence alignments into the corresponding codon alignments. Nucleic Acids Res.

[70] Guindon S (2010). New algorithms and methods to estimate maximum-likelihood phylogenies: assessing the performance of PhyML 3.0. Syst. Biol..

[71] Lefort V, Longueville J-E, Gascuel O (2017). SMS: Smart Model Selection in PhyML. Mol Biol Evol.

[72] Anisimova M, Bielawski JP, Yang Z (2001). Accuracy and power of the likelihood ratio test in detecting adaptive molecular evolution. Mol. Biol. Evol..

[73] Letunic I, Bork P (2016). Interactive tree of life (iTOL) v3: an online tool for the display and annotation of phylogenetic and other trees. Nucleic Acids Res.

[74] Goldman N, Yang Z (1994). A codon-based model of nucleotide substitution for protein-coding DNA sequences. Mol. Biol. Evol..

[75] Nielsen R, Yang Z (1998). Likelihood models for detecting positively selected amino acid sites and applications to the HIV-1 envelope gene. Genetics.

[76] Yang Z (1998). Likelihood ratio tests for detecting positive selection and application to primate lysozyme evolution. Mol. Biol. Evol..

[77] Yang Z, Wong WSW, Nielsen R (2005). Bayes empirical bayes inference of amino acid sites under positive selection. Mol. Biol. Evol..

[78] Vuong QH (1989). Likelihood Ratio Tests for Model Selection and Non-Nested Hypotheses. Econometrica.

[79] Yang Z, Nielsen R, Goldman N, Pedersen AM (2000). Codon-substitution models for heterogeneous selection pressure at amino acid sites. Genetics.

[80] Webb B, Sali A (2016). Comparative Protein Structure Modeling Using MODELLER. Curr Protoc Bioinformatics.

[81] Heo L, Park H, Seok C (2013). GalaxyRefine: Protein structure refinement driven by side-chain repacking. Nucleic Acids Res..

[82] Krieger E (2009). Improving physical realism, stereochemistry, and side-chain accuracy in homology modeling: Four approaches that performed well in CASP8. Proteins.

[83] Chen VB (2010). MolProbity: all-atom structure validation for macromolecular crystallography. Acta Crystallogr D Biol Crystallogr.

[84] Baker NA, Sept D, Joseph S, Holst MJ, McCammon JA (2001). Electrostatics of nanosystems: application to microtubules and the ribosome. Proc. Natl. Acad. Sci. USA.

[85] Dolinsky TJ, Nielsen JE, McCammon JA, Baker NA (2004). PDB2PQR: an automated pipeline for the setup of Poisson-Boltzmann electrostatics calculations. Nucleic Acids Res..

[86] Tian W, Chen C, Lei X, Zhao J, Liang J (2018). CASTp 3.0: computed atlas of surface topography of proteins. Nucleic Acids Res..

[87] Echave J, Spielman SJ, Wilke CO (2016). Causes of evolutionary rate variation among protein sites. Nat. Rev. Genet..

[88] Durrand V (2004). Variations in the sequence and expression of the Plasmodium falciparum chloroquine resistance transporter (Pfcrt) and their relationship to chloroquine resistance *in vitro*. Mol. Biochem. Parasitol..

