# Supplementary material for: Structural and evolutionary analyses of the Plasmodium falciparum chloroquine resistance transporter
Source: Sci Rep. 2020 Mar 16;10:4842. doi: 10.1038/s41598-020-61181-1 (PMC7076037; doi:10.1038/s41598-020-61181-1)
Supplement: Supplementary file 1 — Supplementary Figures and Tables. [file 41598_2020_61181_MOESM1_ESM.docx]

**Supplementary Figures and Tables**

This supplemental file has been provided by the authors to give readers additional information about their work.

Supplement to: **Structural and evolutionary analyses of the *Plasmodium falciparum* chloroquine resistance transporter**

By Romain Coppée, Audrey Sabbagh, Jérôme Clain

**Supplementary Figure S1 ………………………………………………………………………. 2-3**

**Supplementary Figure S2 ………………………………………………………………………… 4**

**Supplementary Figure S3 ………………………………………………………………………… 5**

**Supplementary Figure S4 ………………………………………………………………………… 6**

**Supplementary Figure S5 ………………………………………………………………………… 7**

**Supplementary Figure S6 ………………………………………………………………………… 8**

**Supplementary Figure S7 ………………………………………………………………………… 9**

**Supplementary Figure S8 ………………………………………………………………………… 10**

**Supplementary Figure S9 ………………………………………………………………………… 11**

**Supplementary Figure S10 ……………………………………………………………………….. 12**

**Supplementary Figure S11 ……………………………………………………………………….. 13**

**Supplementary Figure S12 ……………………………………………………………………….. 14**

**Supplementary Figure S13 ……………………………………………………………………….. 15**

**Supplementary Figure S14 ……………………………………………………………………….. 16**

**Supplementary Figure S15 ……………………………………………………………………….. 17**

**Supplementary Table S1 ………………………………………………………………………….. 18**

**Supplementary Table S2 ………………………………………………………………………….. 19**

**Supplementary Table S3 ………………………………………………………………………….. 20**

**Supplementary Table S4 ………………………………………………………………………….. 21**

**Supplementary Table S5 ………………………………………………………………………….. 22**

**Supplementary Table S6 ………………………………………………………………………….. 23**

**Supplementary Table S7 ………………………………………………………………………….. 24**

**Supplementary Table S8 ………………………………………………………………………….. 25**

**Supplementary Table S9 ………………………………………………………………………….. 26**

**Supplementary Table S10 ………………………………………………………..……………….. 27**

**TM 1**

**TM 2**


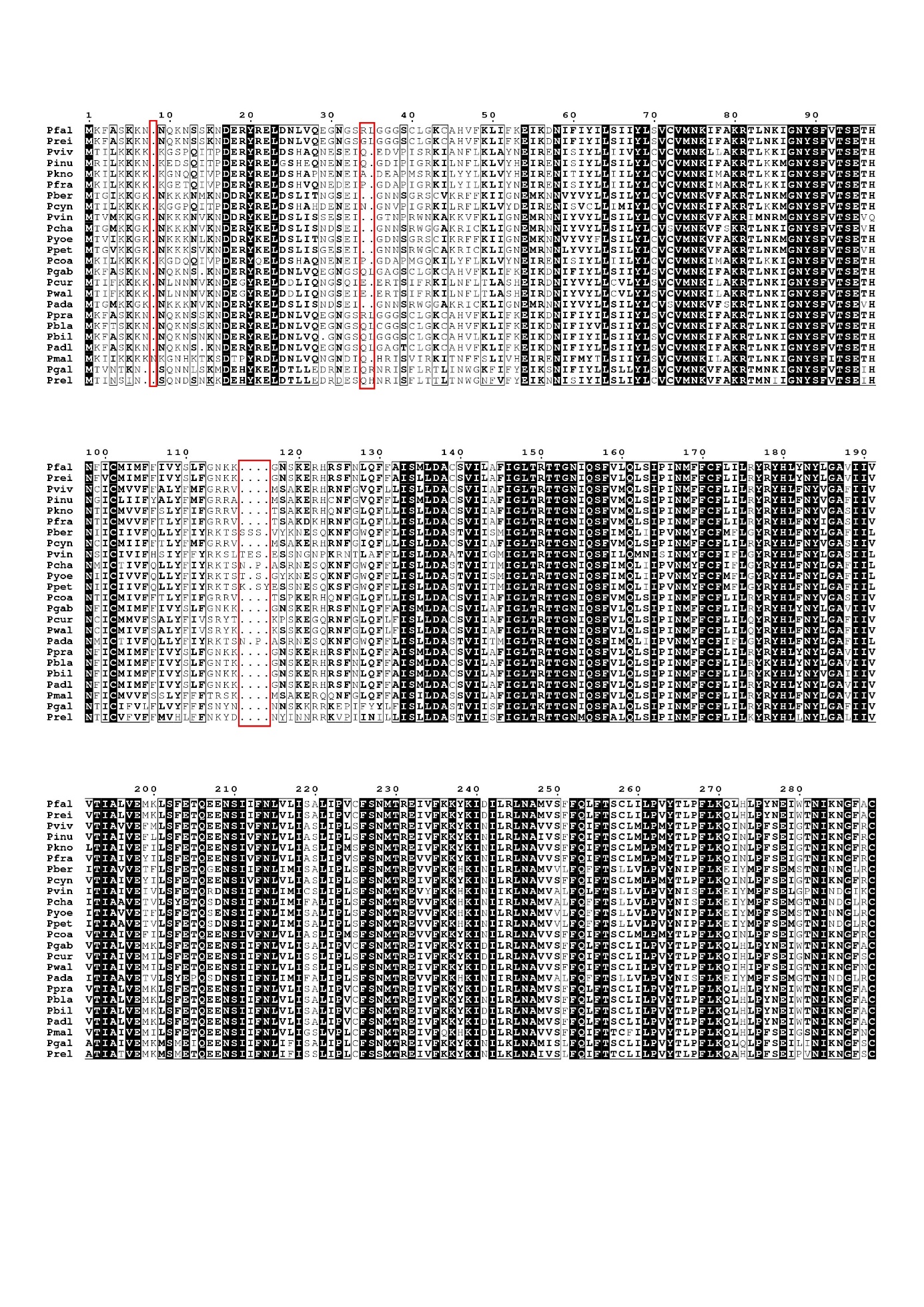


**TM 7**

**TM 6**

**TM 5**

**TM 5**

**TM 4**

**TM 3**

**TM 2**

**TM 10**

**TM 9**

**TM 8**


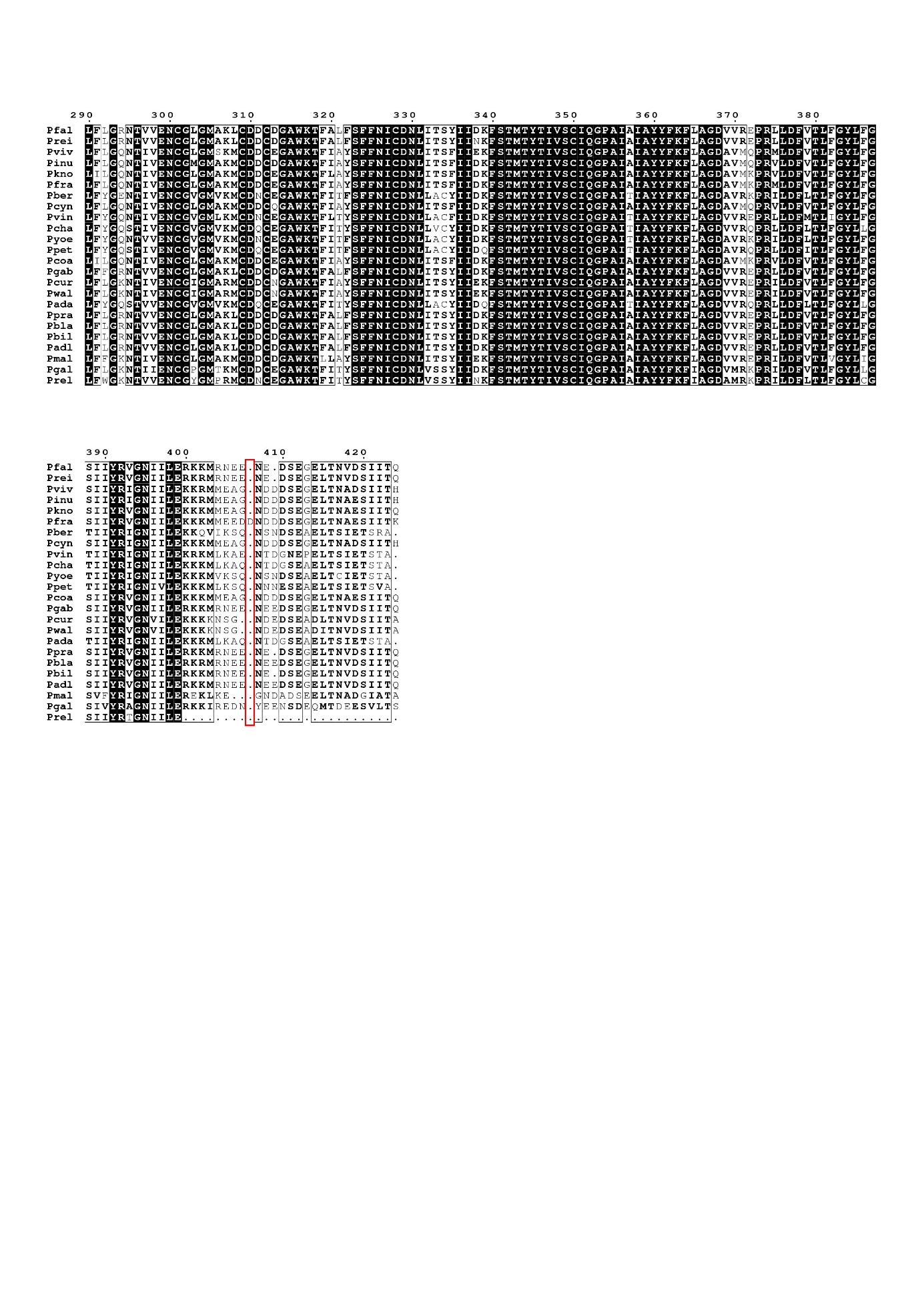


**TM 10**

**Supplementary Figure S1.** **CRT protein multiple sequence alignment.**

The CRT multiple sequence alignment was performed using Mafft version 7. Strictly conserved positions are written in white letters and shaded in black. Highly conserved positions are boxed with black lines and written in black, bolded letters. The level of conservation is based on BLOSUM62 scoring matrix. A four-letter code is used for the name of species (full species names are provided in Supplementary Table S1). The regions of the alignment with highly divergent positions, as well as positions containing gaps in at least 30% of all sequences, were manually removed in some subsequent analyses (red boxes). The multiple sequence alignment was displayed with the ESPript 3.0 server. TMs are indicated above the multiple sequence alignment, delineated using the high-resolution 3D structure of PfCRT (PDB ID: 6ukj).

**
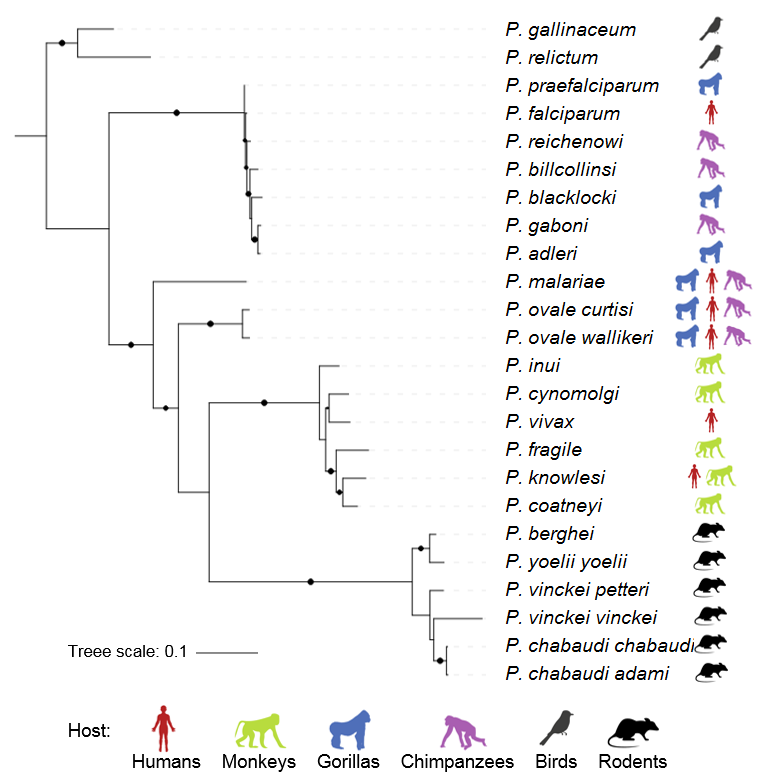
**

**Supplementary Figure 2. Maximum likelihood phylogenetic tree of orthologous *crt* protein-coding sequences from *Plasmodium* species.**

Bootstrap values are shown as black circles (> 90%) using the approximate likelihood ratio aLRT SH-like method. Vertebrate host(s) during intraerythrocytic development is (are) provided for each *Plasmodium* species.

**
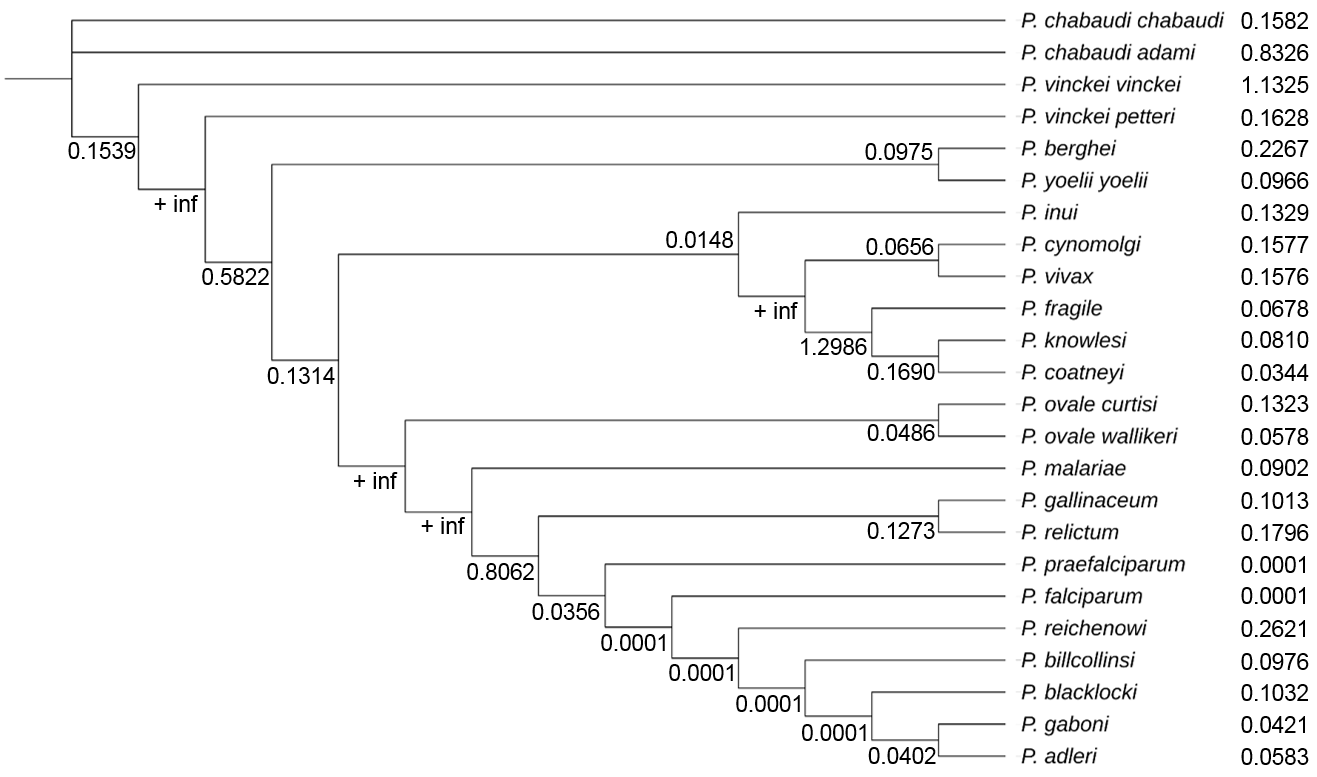
**

**Supplementary Figure S3.** **Branch-specific *ω* values estimated with PAML under the free-ratio (FR) model for the *crt* gene.**

*ω* estimates (*d*_N_/*d*_S_) with “+ inf” values for which the estimation of *d*_S_ was equal to 0.0 (*i.e.* no silent substitutions observed) are designated as extremely low. Branch lengths were ignored for ease of representation. There was suggestive evidence of positive selection for *P. vinckei vinckei* and the common ancestor of *P. fragile*, *P. knowlesi* and *P. coatneyi*. All the remaining lineages evolved under strong purifying selection.


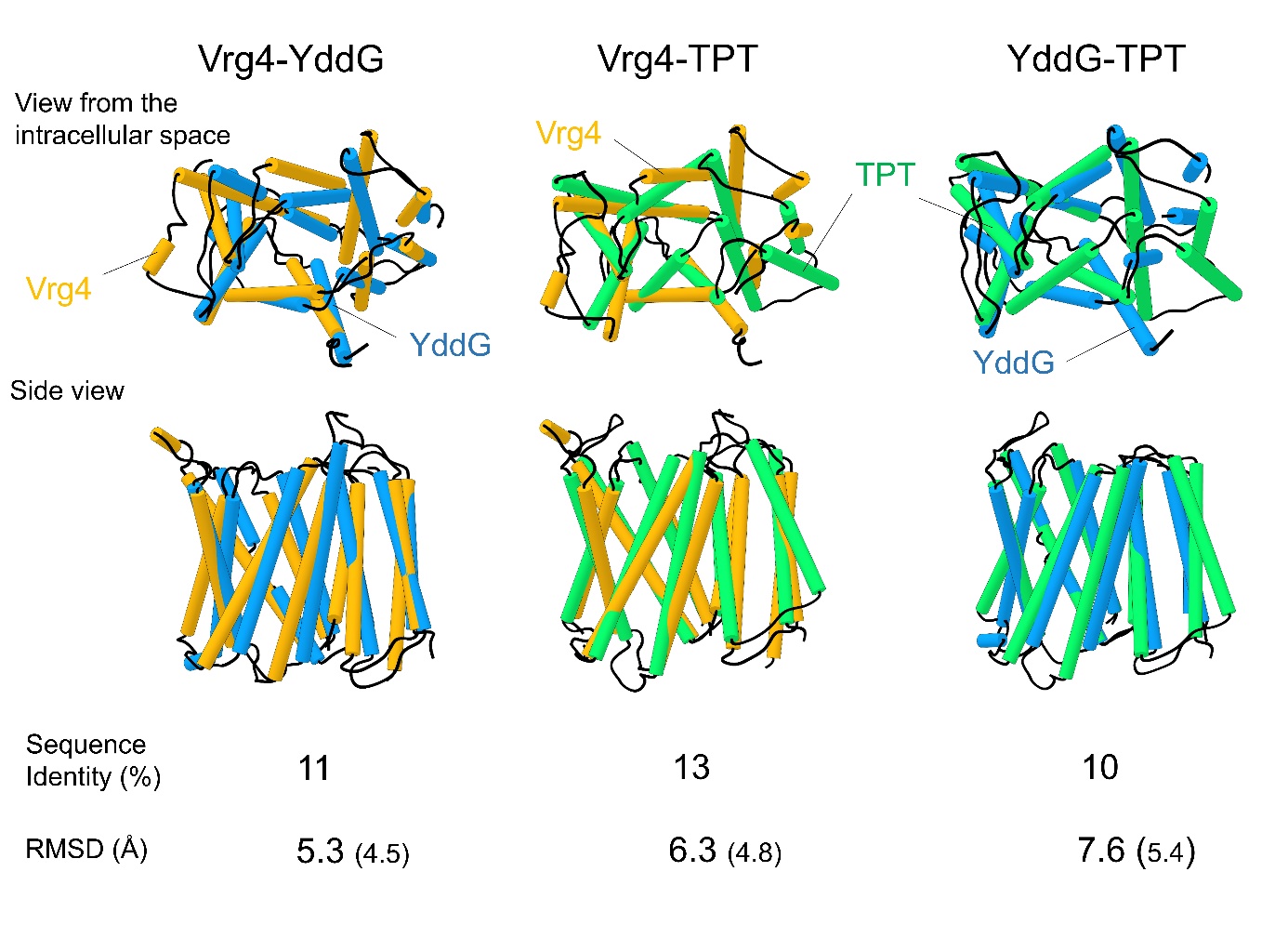


**Supplementary Figure S4.** **Paired superposition of the experimentally-determined structures of the Drug/Metabolite Transporters (DMTs) Vrg4, YddG and TPT.**

Each pair of structures was aligned using the *MatchMaker* function implemented in UCSF Chimera. Structures are shown from the intracellular face (*top* structures) and from the side (*bottom* structures). Helices are displayed as cylinders. Vrg4 (PDB ID: 5oge), YddG (PDB ID: 5i20) and TPT (PDB ID: 5y79) are colored in orange, blue and green, respectively. These proteins share a similar fold, despite a sequence identity < 15% (see Supplementary Table S5). The RMSD in Å and the % of sequence identity are indicated under each set of superposed structures. The pairwise sequence identities were calculated using AlignME. RMSD values were computed using Cɑ atoms (values in parentheses are those obtained when TMs-connecting loops were excluded).


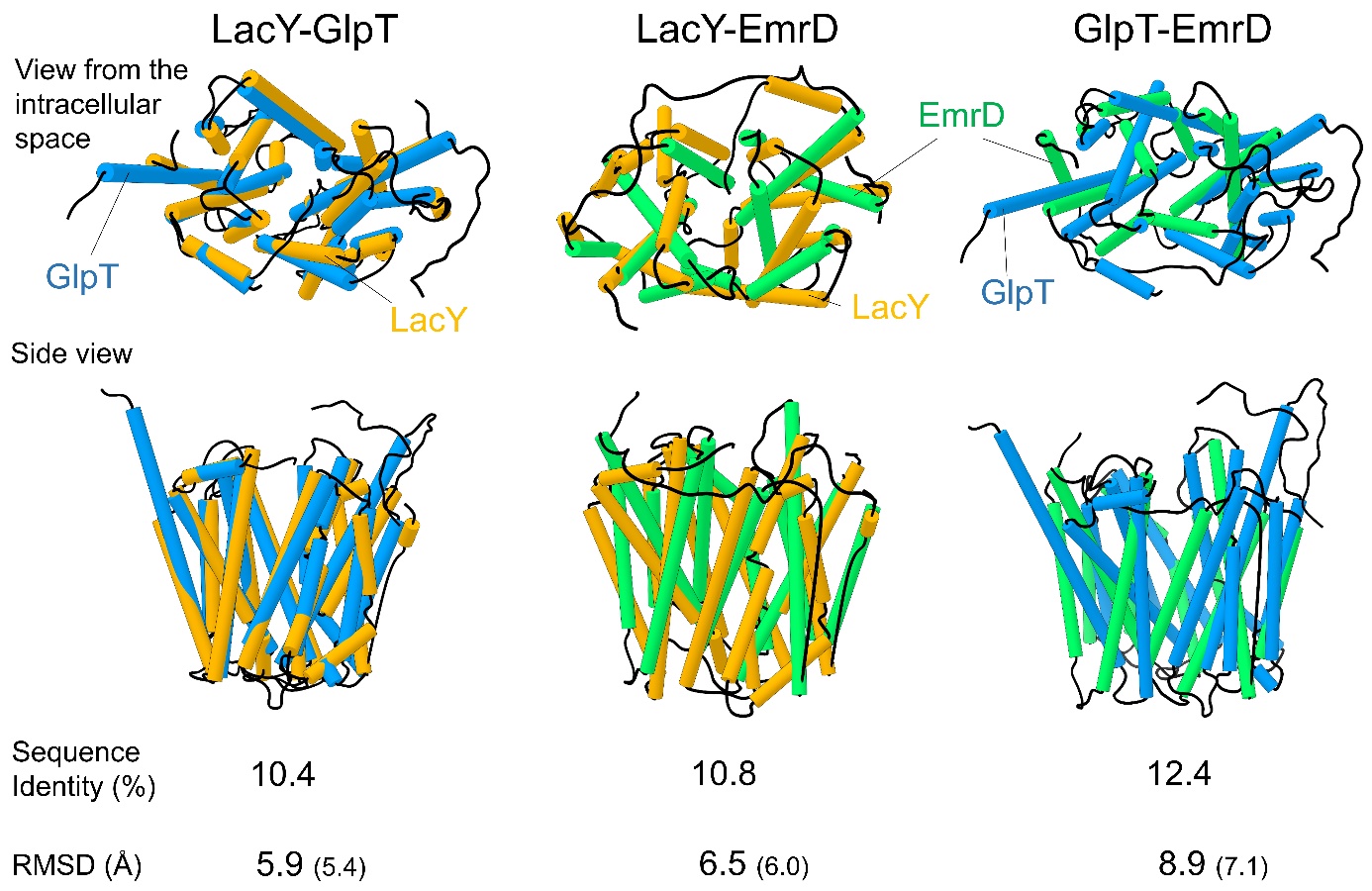


**Supplementary Figure S5.** **Paired superposition of the experimentally-determined structures of the Major Facilitator Superfamily (MFS) LacY, GlpT and EmrD.**

Each pair of structures was aligned using the *MatchMaker* function implemented in UCSF Chimera. Structures are shown from the intracellular face (*top* structures) and from the side (*bottom* structures). Helices are displayed as cylinders. LacY (PDB ID: 2y5y), GlpT (PDB ID: 1pw4) and EmrD (PDB ID: 2gfp) are colored in orange, blue and green, respectively. These proteins share a similar fold, despite a sequence identity < 15%. The RMSD in Å and the % of sequence identity are indicated under each set of superposed structures. The pairwise sequence identities were calculated using AlignME. RMSD values were computed using Cɑ atoms (values in parentheses are those obtained when TMs-connecting loops were excluded).

*** * * * * ** * * ***

--------- TM 1 -------- TM 2 .

**PfCRT** K**E**IKDN**I**FIYI**L**SII**YL**S**V**C**VMNK**IFA**KR**TLNKI-**GNYSF**V**TSE**TH**N**FI**C**MIMFFIVYSL

**Vrg4** --------GPISILS**Y**CGSSILMTVTNKFVVNLKDFNMNFVMLFVQSLVCTITLII-LRI

--------- TM 1 -------- TM 2 .

*** * ** * * * * ***

------- ---------- TM 3 -- TM 4-----

**PfCRT** FGNKKGNSKERHRSFNLQFFA**IS**M**LDA**CS**VI**LAF**IGLT**R**TTGN**I**QSF**VL**Q**LS**I**PI**NM**F**FC**

**Vrg4** LGYA-KFRSL-NKTDAKNWFPISFLLVLMIYTSSKALQYLAVPI**Y**TIF**K**NLTIILIAYGE

------------- TM 3 -- TM 4-----

*** ***

----- TM 5 --------------------- ------

**PfCRT** **F**LI**L**R**Y**R**YHL**Y**NY**L**GA**V**II**VV**TIA**L**VE**MKL-**S**F**E**T**Q**----------EE--**NSI**I**FNL**VL**I**

**Vrg4** VLFFGGSVTSMELSSFLLMVLSSVVATWGDQQAVAAKAASLAEGAAGAVASFNPGYFWMF

----- TM 5 ------------------------- ------

**#**

*** * * ***

----TM 6---------- --- TM 7 --------------

**PfCRT** SA**L**I**P**VC**FS**N**MT**R**E**IV**F**K**K**Y**KI**D**I**LR**LNA**MVSF**FQ**L**FT**SCLI**LP**V**Y**TLP**FGAWKT**FALF**S**

**Vrg4** TNCITSALFVLIMRKRIKLTNFKDFDTMFY**NN**VLALPILLLFSFC-----DSLTAMIISG

----TM 6---------- ----- TM 7 ----- ---------

*** ** * * * ***

----TM 8 - TM 9--------- ---------- --------

**PfCRT** **FFNICDNL**ITSY**II**DK**FSTMTYTIVSCIQGPAI**A**IAYYFKF**L**AGD**VVRE**PR**L**LDF**V**TL**F**G**

**Vrg4** VASVGI**S**YC**S**GWCVRVTSSTT**Y**SMV**G**ALN-**K**LPIALSGLIF-FDA---PRNFLSILSIFI

----TM 8 - TM 9--------- ---------- --------

***** ***

--TM 10------------

**PfCRT** **YL**F**G**SII**YR**V**GN**II**LE**RKK

**Vrg4** GFLSGIIYAVAKQKKQQAQ

--TM 10---------- -

**Supplementary Figure S6. Sequence alignment of PfCRT with Vrg4 template used to predict PfCRT^IF^.**

In red, strictly conserved CRT amino acid position across *Plasmodium* species.

**P**osition belonging to the top 10% most conserved sites of PfCRT, as inferred by FuncPatch, are highlighted with a yellow background.

**A**mino acid position from template sequence involved in substrate binding are bolded and underlined.

*****: identical amino acid between PfCRT and template sequences.

***:** amino acid predicted to be important to align based on profile(CRT)-profile(DMT) alignment.

-- --: secondary structure is indicated above PfCRT and below Vgr4 template (PDB IDE: 5oge, chain A). Grey shading and dash signs indicate helices and loops respectively. For PfCRT, secondary structures were established using the subsequent produced PfCRT^IF^ model.

#: indicates the location of positions 269 to 313 (corresponding to the long vacuolar loop of PfCRT) that were removed because of insufficient amino acid coverage in DMT templates.

*** ***** * * * ***

- TM 1 --- TM 2 ----

**PfCRT** KDN**I**FIYI**L**SII**YL**S**V**C**VMNK**IFA**KR**TLNKI**GNYSF**V**TSE**TH**N**FI**C**MIMFFIVYSLFGNK

**TPT** VHTLKVGFYFFLWYFFNFIFNIANKRTLNMWK-YPWVLSTIQLGVGALYCTFLW-VLGLR

------ TM 1 ---- TM 2 ---

*** * * ***

--------------- TM 3 -- TM 4 ]

**PfCRT** KGNSKERHRSFNLQFFA**IS**M**LDA**CS**VI**LAF**IGLT**R**TTGN**I**QSF**VL**Q**LS**I**PI**NM**F**FCF**LI**L**

**TPT** -TKPNVSKK-LIKALIWPSLGHTLG**H**AATCMSFSLVAISFTHVV**K**SAEPVFGAVGSALVL

------- TM 3 -- TM 4 ]

*** ** * ** ** * * ***

----- TM 5 ------- TM 6---------

**PfCRT** R**Y**R**YHL**Y**NY**L**GA**V**II**VV**TIA**L**VE**MKL**S**F**E**T**Q**EE**NSI**I**FNL**VL**I**SA**L**I**P**VC**FS**N**MT**R**E**IV**F**

**TPT** GEFFHPLTYLTLVPIVSGVALSAA-----TELTFTWTGFITAMISNVAFVTRNITSKFTM

----- TM 5 ----- TM 6---------

**#**

*** * ** * * ***

-- ----------- TM 7 ---- TM 8 -----

**PfCRT** K**K**Y----**KI**D**I**LR**LNA**MVSF**FQ**L**FT**SCLI**LP**V**Y**TLP**FGAWKT**FALF**SFFNICDNL**ITSY**I**

**TPT** VDFKNEKTLIAQNTYALITIISFFM---ELPFAL-LMKLFGSIMFCSLFYHL**Y**NEVSYLC

-- ------- TM 7 TM 8------

*** * * * * * * * * ***

-- - TM 9 -------------- TM 10-----

**PfCRT** **I**DK**FSTMTYTIVSCIQGPAI**A**IAYYFKF**L**AGD**VVRE**PR**L**LDF**V**TL**F**GYL**F**G**SII**YR**V**GN**I

**TPT** LDNVSPVSFSIGNTI**KR**VII-IFGSILVF-RT---PVTRLNFIGSTIAIIGTMLYSLAKA

-- -- TM 9 --------- TM 10-----

*****

-

**PfCRT** I**L**

**TPT** KL

-

**Supplementary Figure S7.** **Sequence alignment of PfCRT with TPT template used to predict the PfCRT^OC^.**

In red, strictly conserved CRT amino acid position across *Plasmodium* species.

**P**osition belonging to the top 10% most conserved sites of PfCRT, as inferred by FuncPatch, are highlighted with a yellow background.

**A**mino acid position from template sequence involved in substrate binding are bolded and underlined.

*****: identical amino acid between PfCRT and template sequences.

***:** amino acid predicted to be important to align based on profile(CRT)-profile(DMT) alignment.

-- --: secondary structure is indicated above PfCRT and below TPT template (PDB IDE: 5y79, chain A). Grey shading and dash signs indicate helices and loops respectively. For PfCRT, secondary structures were established using the subsequent produced PfCRT^OC^ model.

#: indicates the location of positions 269 to 313 (corresponding to the long vacuolar loop of PfCRT) that were removed because of insufficient amino acid coverage in DMT templates.


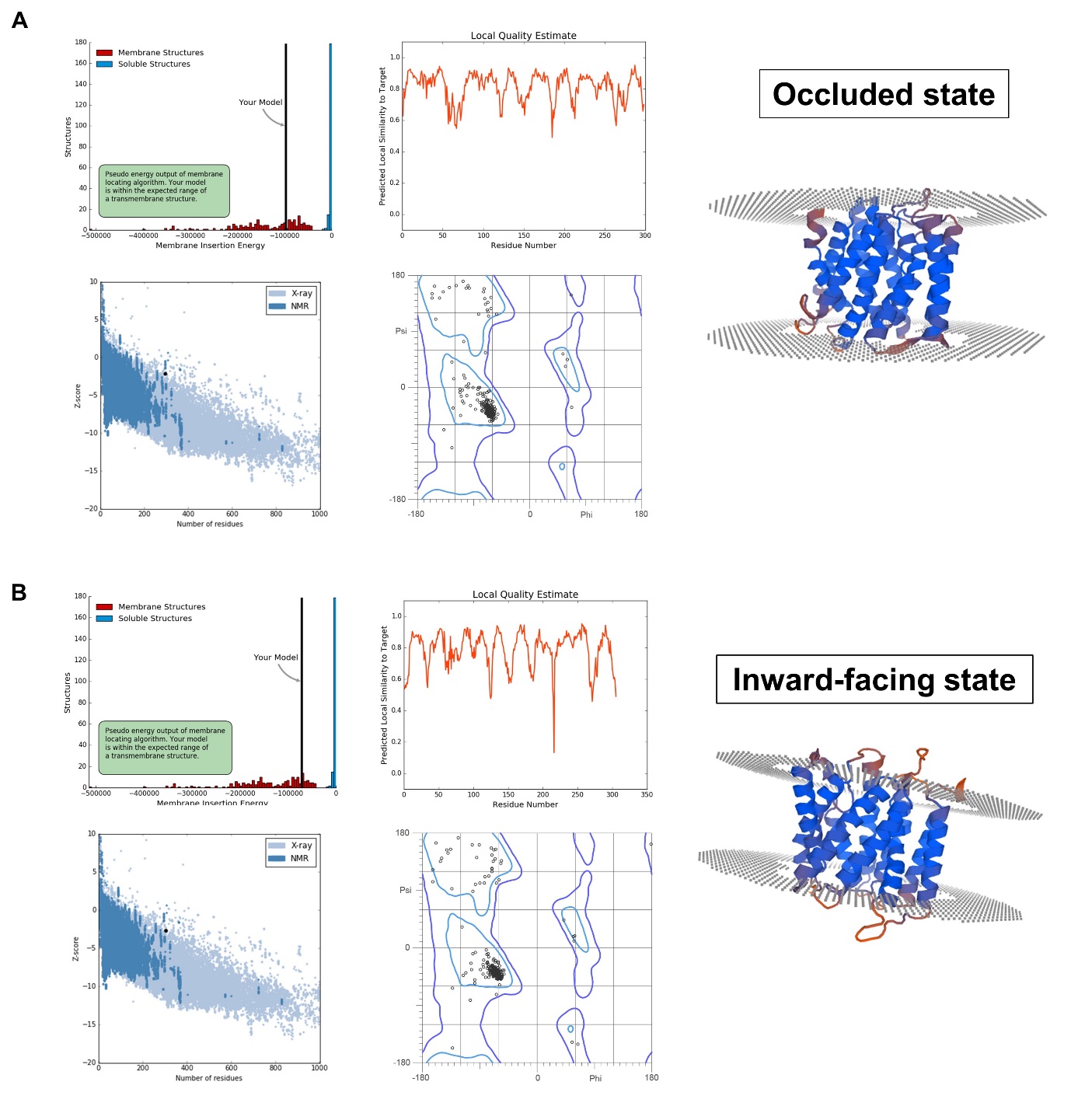


**Supplementary Figure S8. Validation of the refined, energy-minimized PfCRT^IF^ and PfCRT^OC^ models obtained by homology modeling.**

General and transmembrane-specialized 3D quality metrics of the PfCRT^OC^ (**A**) and PfCRT^IF^ (**B**) models. *Upper plots*: QMEANBrane analyses showed that the pseudo-energy of PfCRT models (*left* histograms) fell within the range of expected pseudo-energies in transmembrane structures. The graph (*right*) exhibits the per-residue local quality estimate for PfCRT models. As shown in the 3D structures, residues located in TMs had high quality estimates (most of them ranging from 0.8 to 1, colored in blue in the 3D structure), while the loops were associated with lower scores (colored in orange in 3D structure). *Bottom plots*: ProSA II showed the distribution of Z-scores calculated for all experimentally determined protein chains available in the Protein Data Bank (*left* plot). PfCRT models (represented as a black circle in the plots) were located in the range of structured resolved with NMR method. The Ramachandran plot from MolProbity (*right* plot) showed that most residues of PfCRT models had favorable ψ and ϕ angles, without outliers.


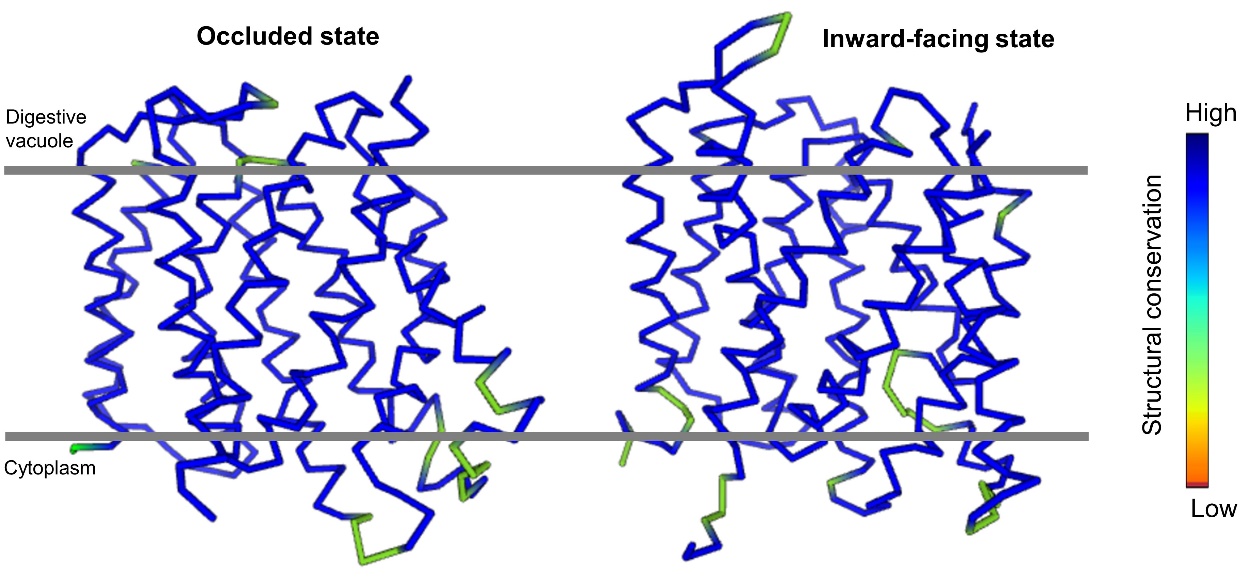


**Supplementary Figure S9. Structural conservation profile between the PfCRT models and the high-resolution PfCRT^cryo-EM^ structure.**

The PfCRT^OC^ and PfCRT^IF^ models are shown using Cɑ trace, from the side. Conservation profiles were generated using the Dali server. TMs were highly conserved, whilst some TMs-connecting loops indicated a lower structural conservation.


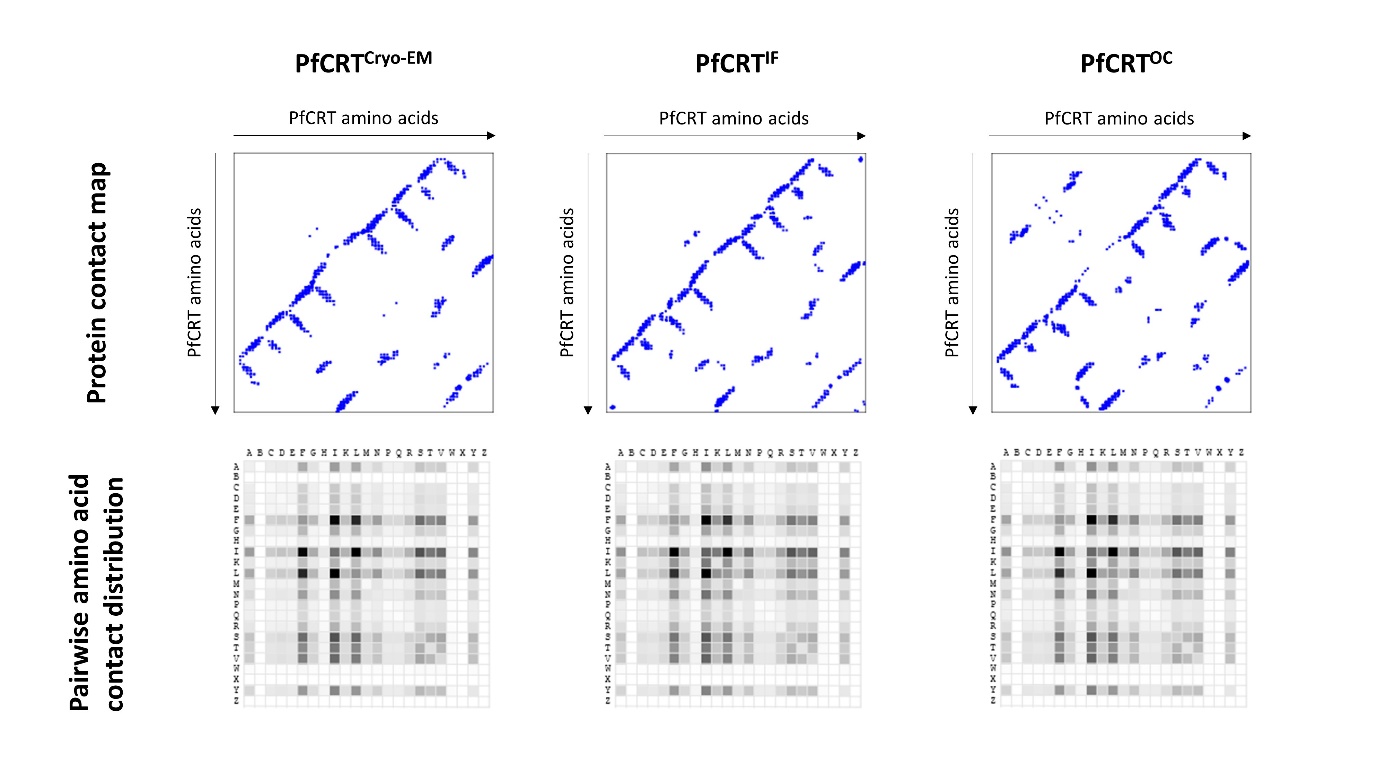


**Supplementary Figure S10. Cɑ-based contact maps and pairwise amino acid contact distributions of the PfCRT models and PfCRT^cryo-EM^.**

On the protein contact maps, a blue point corresponds to a pair of PfCRT amino acid sites. The main differences between the PfCRT^cryo-EM^ and PfCRT^IF^ maps correspond to contact involving TMs-connecting loops, whereas the main differences between the PfCRT^IF^ and PfCRT^OC^ maps correspond to positions involved in the switch of the vacuolar half of TM 3 and TM 4 at the entrance of the cavity (vacuolar gate). On the pairwise amino acid contact distributions, the darker the square, the higher the proportion of the paired amino acids making contact in the PfCRT tertiary structure. The plots were produced using the CMWeb server (default parameters). The letters correspond to the international one-letter amino acid code. B: aspartic acid or asparagine, Z: glutamic acid or glutamine, X: any amino acid, J: leucine or isoleucine.


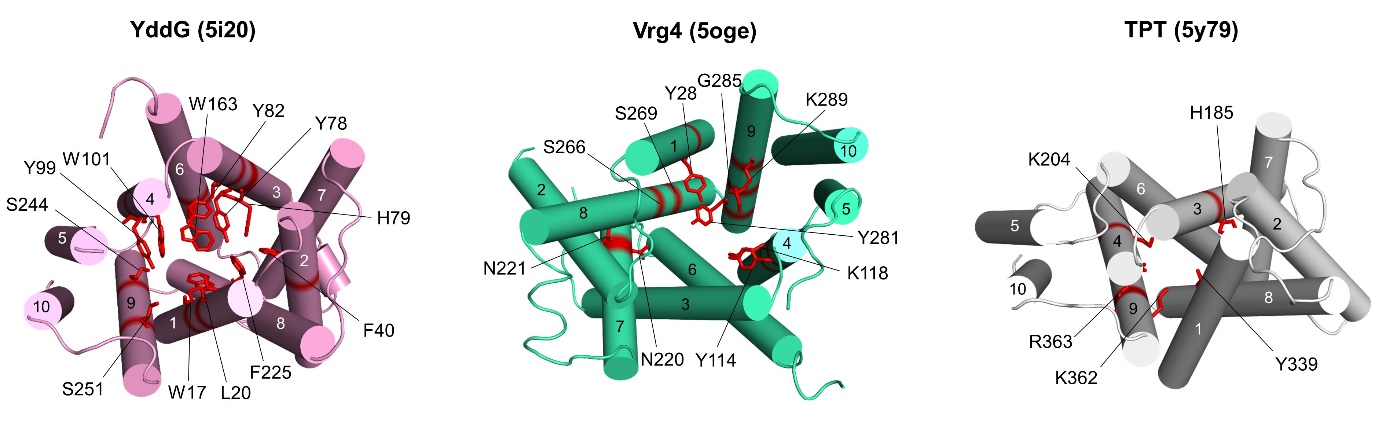


**Supplementary Figure S11. Amino acid sites of the template transporter proteins that are known to bind physiological substrate(s).**

The key amino acid sites binding the substrate(s) of three 10 TMs-containing DMT transporters are shown in red color as sticks. The overall structures are shown as cartoon from the intracellular face which corresponds to the vacuolar face for PfCRT. The name of the proteins and corresponding PDB identifiers (within brackets) are provided above each structure.

**
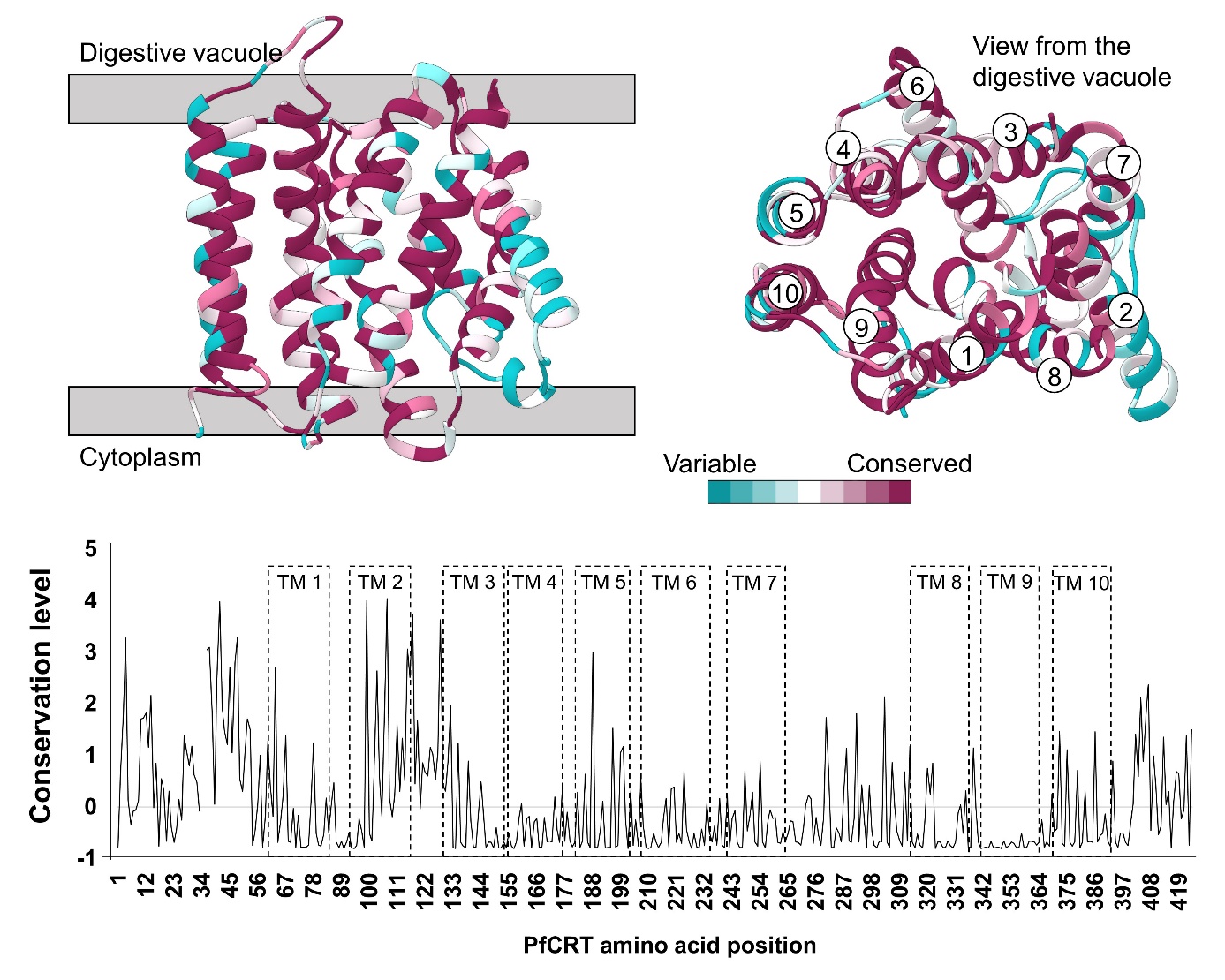
**

**Supplementary Figure S12. Conservation level per PfCRT amino acid site using the Consurf web server.**

Similarly to PAML and FuncPatch methods, the Consurf web server estimates a level of conservation per codon/amino acid site using a multiple sequence alignment and the corresponding phylogenetic tree. A tertiary structure is also provided and serves to visualize the distribution of the conservation levels, but is not included to check the spatial correlation of site-specific substitution rates attributed to tertiary structure like FuncPatch. In the top panel, the PfCRT^IF^ model is shown as cartoon. Cyan corresponds to variable amino acid sites, while those in purple are much conserved as categorized by Consurf. The label of TMs are indicated. The bottom panel shows the level of conservation along the PfCRT amino acid sequence. The location of TMs is indicated as dotted boxes. The TM 9 is the more conserved TM as observed also using FuncPatch.

**
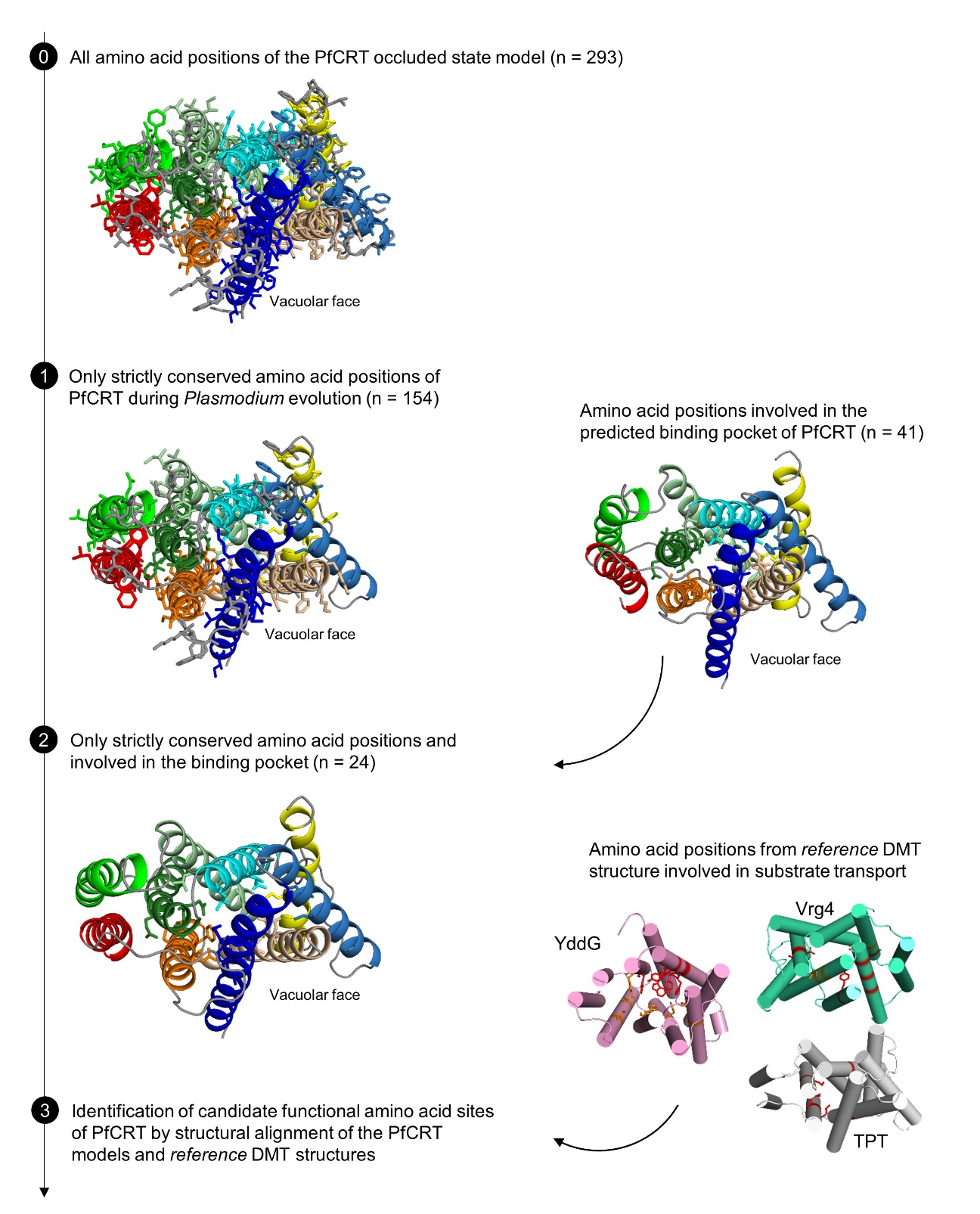
**

**Supplementary Figure S13. Filtering of PfCRT amino acid sites for the identification of candidate functional sites.** The PfCRT^OC^ model is colored according to TMs. YddG, Vrg4 and TPT proteins are colored in pink, blue-green and grey, respectively. Functional amino acid sites of these proteins are shown in stick and colored in red. Starting with the PfCRT^OC^ model covering 293 amino acid positions of the PfCRT sequence, only the strictly conserved amino acid sites at the *Plasmodium* scale were selected (n = 154). Meanwhile, the amino acid sites involved in the architecture of the predicted binding pocket were selected (n = 41). Among these sites, those that are strictly conserved during *Plasmodium* evolution were kept (n = 24). Finally, the PfCRT^OC^ and PfCRT^IF^ models were structurally aligned with the different DMT template structures used in this study (*i.e.* YddG, Vrg4 and TPT proteins). We then focused on the subset of PfCRT amino acid sites that structurally aligned with experimentally-tested and validated amino acid sites of the reference DMT proteins (shown in the Supplementary Figure S11).


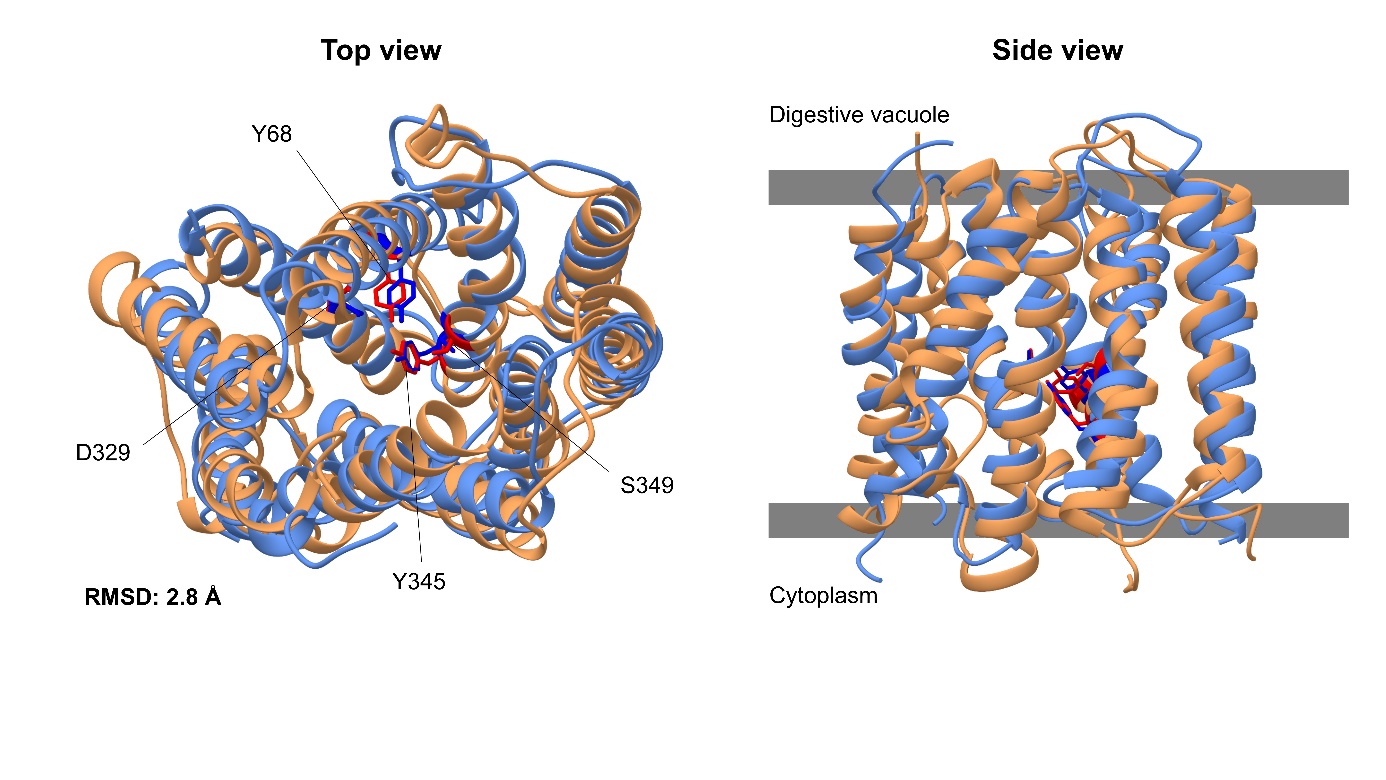


**View from the digestive vacuole**

**Supplementary Figure S14.** **Paired superposition of the high-resolution 3D PfCRT structure determined by cryo-EM (PfCRT^cryo-EM^) and our PfCRT^IF^ model.**

The two structures were aligned using the *MatchMaker* function implemented in UCSF Chimera. Structures are shown from the digestive vacuole (*left* structures) and from the side (*right* structures). The experimentally-determined PfCRT tertiary structure (PDB ID: 6ukj) and our PfCRT^IF^ model are colored in blue and orange, respectively. The RMSD between the two structures was computed using all Cɑ atoms and considering TMs-connecting loops. When TMs-connecting loops were excluded, the RMSD was 2.4 Å. The four candidate functional sites of PfCRT that we propose have the same side-chain orientation than in PfCRT^cryo-EM^. These positions are shown as red and blue stick in PfCRT^IF^ model and PfCRT^cryo-EM^, respectively.

**
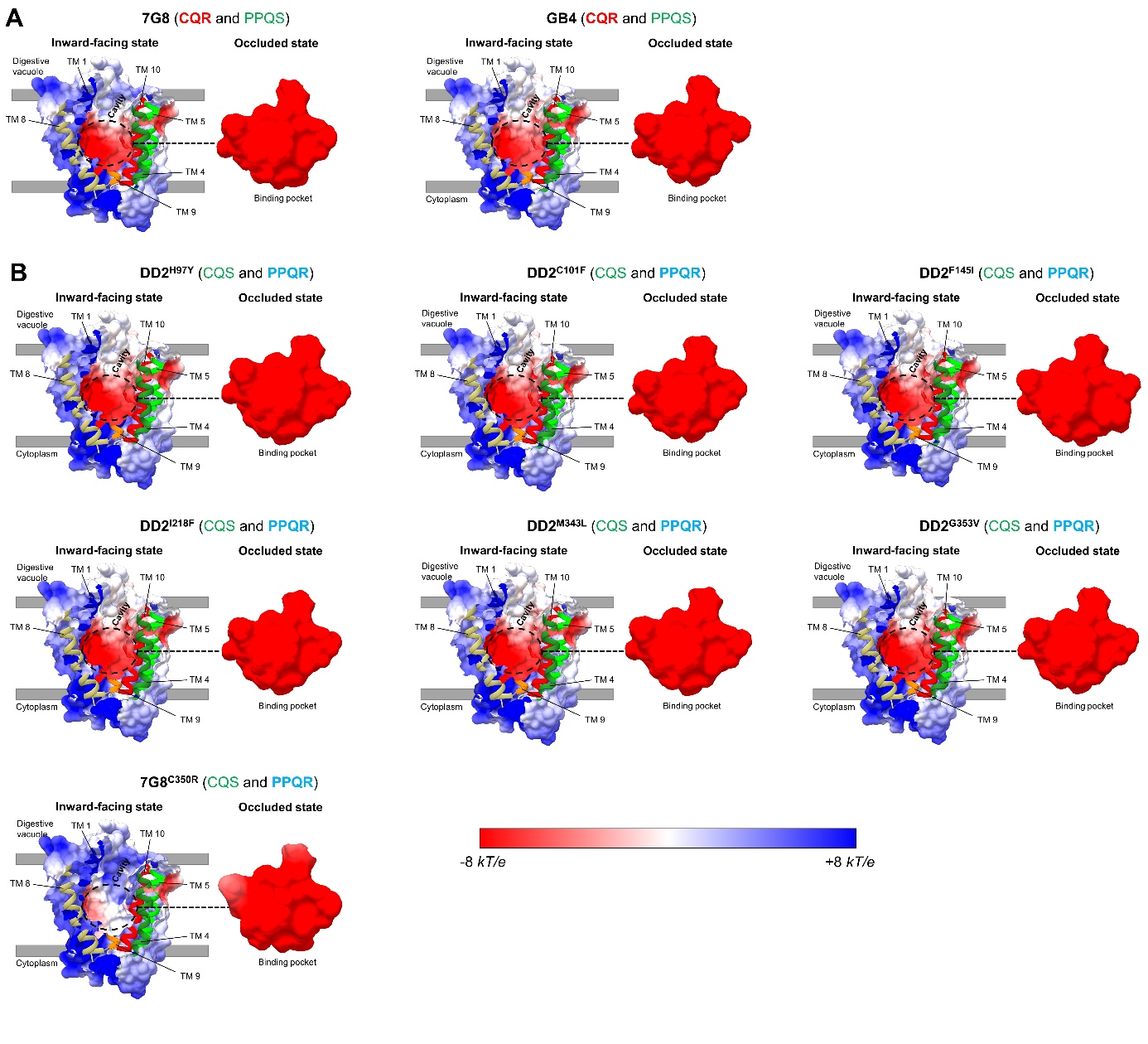
**

**Supplementary Figure S15. Electrostatic surface potential highlighting the cavity and binding pocket of PfCRT in a CQR or PPQR context.**

The electrostatic surface potential is displayed for the CQR haplotypes 7G8 and GB4 (**A**) and for seven other PPQR mutant structures (**B**). For each structure are indicated: the TMs (colored according to Figure 1A and 1B), the drug phenotype (CQR, chloroquine resistance; CQS, chloroquine sensitivity; PPQR, piperaquine resistance; PPQS, piperaquine sensitivity). Structures are shown from the side to highlight the cavity in PfCRT^IF^ (*left* structures), in addition to a zoom on the binding pocket from PfCRT^OC^ (*right* structures). Note that only one viewing angle of the PfCRT cavity is shown here, but any other viewing angle exhibited the same electrostatic surface potential.

**Supplementary Table S1. Description of CRT orthologous sequences used in this study.**

| **Species** | **Abbreviation** | **Description** | **Accession number** | **Source** | **Length (AA)** |
| --- | --- | --- | --- | --- | --- |
| *Plasmodium falciparum* | Pfal | chloroquine resistance transporter | PF3D7_0709000 | PlasmoDB | 424 |
| *Plasmodium reichenowi* | Prei | chloroquine resistance transporter | PRCDC_0707200 | PlasmoDB | 424 |
| *Plasmodium vivax* | Pviv | chloroquine resistance transporter | PVX_087980 | PlasmoDB | 424 |
| *Plasmodium inui* | Pinu | — | — | — | 424 |
| *Plasmodium knowlesi* | Pkno | chloroquine resistance transporter | PKNH_0107600 | PlasmoDB | 424 |
| *Plasmodium fragile* | Pfra | hypothetical protein | AK88_01012 | PlasmoDB | 425 |
| *Plasmodium berghei* | Pber | chloroquine resistance transporter | PBANKA_1219500 | PlasmoDB | 425 |
| *Plasmodium cynomolgi* | Pcyn | chloroquine resistance transporter | PCYB_011610 | PlasmoDB | 424 |
| *Plasmodium vinckei vinckei* | Pvin | chloroquine resistance transporter | YYE_01536 | PlasmoDB | 425 |
| *Plasmodium chabaudi chabaudi* | Pcha | chloroquine resistance transporter | PCHAS_1220200 | PlasmoDB | 424 |
| *Plasmodium yoelii yoelii* | Pyoe | chloroquine resistance transporter | PY17X_1222700 | PlasmoDB | 424 |
| *Plasmodium vinckei petteri* | Ppet | chloroquine resistance transporter | YYG_02380 | PlasmoDB | 425 |
| *Plasmodium coatneyi* | Pcoa | chloroquine resistance transporter | PCOAH_00000850 | PlasmoDB | 424 |
| *Plasmodium gaboni* | Pgab | chloroquine resistance transporter | PGSY75_0709000 | PlasmoDB | 424 |
| *Plasmodium malariae* | Pmal | chloroquine resistance transporter | PmUG01_01020700 | PlasmoDB | 423 |
| *Plasmodium ovale curtisi* | Pcur | chloroquine resistance transporter | PocGH01_01016900 | PlasmoDB | 423 |
| *Plasmodium ovale wallikeri* | Pwal | chloroquine resistance transporter | POVWA2_000940 | PlasmoDB | 423 |
| *Plasmodium gallinaceum* | Pgal | chloroquine resistance transporter | PGAL8A_00049200 | PlasmoDB | 424 |
| *Plasmodium relictum* | Prel | chloroquine resistance transporter | PRELSG_0107100 | PlasmoDB | 398 |
| *Plasmodium chabaudi adami* | Pada | chloroquine resistance transporter | SCN62053.1 | GenBank | 424 |
| *Plasmodium adleri* | Padl | chloroquine resistance transporter | PADL01_0707800 | PlasmoDB | 424 |
| *Plasmodium billcollinsi* | Pbill | chloroquine resistance transporter | PBILCG01_0708300 | PlasmoDB | 423 |
| *Plasmodium blacklocki* | Pbla | chloroquine resistance transporter | PBLACG01_0708200 | PlasmoDB | 425 |
| *Plasmodium praefalciparum* | Ppra | chloroquine resistance transporter | PPRFG01_0708600 | PlasmoDB | 424 |

Note – For *P. ovale* spp. and *P. inui* sequences, we manually performed exon skipping to reconstruct the full cDNA and amino acid sequences. Only the bird-infecting *P. relictum* species seemed incomplete with an absence of about twenty amino acid positions in the C-terminal extremity of CRT.

**Supplementary Table S2. Results of PAML analyses for the *crt* gene.**

| **Model** | **ℓ** | **Parameters** | **κ** | **Positively Selected Codons** |
| --- | --- | --- | --- | --- |
| free-ratio | -10,436.32 | *ω* can vary between branches | 2.31 | Not allowed |
| M0 | -10,566.42 | *ω* = 0.119 | 2.27 | Not allowed |
| M1a | -10,383.20 | *ω_0_* = 0.077, p_0_ = 0.817  *ω_1_* = 1.000, p_1_ = 0.183 | 2.72 | Not allowed |
| M2a | -10,383.20 | *ω_0_* = 0.077, p_0_ = 0.817  *ω_1_* = 1.000, p_1_ = 0.056  *ω_2_* = 1.000, p_2_ = 0.127 | 2.72 | None detected |
| M3  (*k* = 4) | -10,254.80 | *ω_0_* = 0.000, p_0_ = 0.299  *ω_1_* = 0.054, p_1_ = 0.330  *ω_2_* = 0.241, p_2_ = 0.266  *ω_3_* = 0.603, p_3_ = 0.105 | 2.41 | None detected |
| M7 | -10,255.75 | *β*(0.357, 2.047)  *ω* ≤ 1.000, p = 1.000 | 2.41 | Not allowed |
| M8 | -10,255.75 | *β*(0.357, 2.047)  *ω* ≤ 1.000, p_0_ = 1.000 | 2.41 | None detected |

Note – *ω*, *d*_N_/*d*_S_ ratio; *p_n_*, proportion of codon sites in the site class *ω_n_*; ℓ, log-likelihood value (the larger ℓ, the better fit the model is); κ, transition/transversion rate ratio; *β*(*p*, *q*), shape parameters for the *β*-distribution of *ω*. Candidate codon sites for positive selection are identified using the Bayes empirical Bayes (BEB) inference for models M2a and M8, and the Naïve empirical Bayes (NEB) inference for model M3.

**Supplementary Table S3. Ranking of the 10 best templates recommended by Phyre2 to model a PfCRT tertiary structure**

| **PDB structure ^a^** | ***Confidence* criterion ^b^ (%)** | **Coverage ^c^ (%)** | **Number of helices ^d^** | **TCDB ^e^** |
| --- | --- | --- | --- | --- |
| 6I1R ^f^ | 99.5 | 79 | 10 | 2.A.7 |
| **5Y79** | 99.5 | 82 | 10 | 2.A.7 |
| **5OGE** | 99.4 | 79 | 10 | 2.A.7 |
| 5I20 | 98.7 | 80 | 10 | 2.A.7 |
| 1S7B | 51.0 | 20 | 8 | 2.A.7 |
| 3MLG | 41.8 | 12 | 4 | — |
| 2I5N | 39.3 | 7 | 12 | 3.E.2 |
| 2KNC | 34.3 | 11 | 2 | — |
| 5FIY | 32.0 | 4 | Coiled-coil | — |
| 2N1P | 30.0 | 5 | 1 | — |

Note – ^a^ Templates used to model the PfCRT tertiary structure in the present study are written in bold. ^b^ The *confidence* criterion represents the probability that the match between the query sequence (PfCRT) and the template arises from a true relationship and not by chance alone. ^c^ The coverage indicates the percentage of the PfCRT sequence covered by the template amino acid sequence. ^d^ Number of TMs in the template structure. ^e^ The Transporter Classification Database (TCDB) details a comprehensive and approved classification system for membrane transport proteins (<http://www.tcdb.org/>). Each family and sub-family has a distinct identifier. ^f^ The 6I1R structure has been released after we finalized the modeling of PfCRT tertiary structure in different conformational states and during the revision of the manuscript.

**Supplementary Table S4. Ranking of the 10 best templates recommended by HHpred to model a PfCRT tertiary structure**

| **PDB structure ^a^** | ***Confidence* criterion ^b^ (%)** | **E-value ^c^** | **Number of helices ^d^** | **TCDB ^e^** |
| --- | --- | --- | --- | --- |
| **5Y79** | 99.9 | 1.7 e^-23^ | 10 | 2.A.7 |
| **5OGE** | 99.9 | 1.9 e^-23^ | 10 | 2.A.7 |
| 6I1R ^f^ | 99.9 | 9.1 e^-23^ | 10 | 2.A.7 |
| 5I20 | 99.9 | 5.4 e^-21^ | 10 | 2.A.7 |
| 4M64 | 81.8 | 60 | 12 | 2.A.2 |
| 4TPH | 75.2 | 110 | 14 | 2.A.17 |
| 5AEZ | 63.7 | 210 | 11 | 1.A.11 |
| 6C14 | 48.1 | 250 | 5 | 1.A.82 |
| 2N5S | 46.9 | 52 | 2 | — |
| 6E9N | 46.6 | 350 | 12 | 2.A.1 |

Note – ^a^ Templates used to model the PfCRT tertiary structure in the present study are written in bold. ^b^ The *confidence* criteria represents the probability that the match between the query sequence (PfCRT) and the template arises from a true relationship and not by chance alone. ^c^ The E-value for a sequence match is the expected number of false positives per database search with a score at least as good as the score of this sequence match. ^d^ Number of TMs in the template structure. ^e^ The Transporter Classification Database (TCDB) details a comprehensive and approved classification system for membrane transport proteins (<http://www.tcdb.org/>). Each family and sub-family has a distinctive identifier. ^f^ The 6I1R structure has been solved after we finalized the modeling of PfCRT tertiary structure in different conformational states and during the revision of the manuscript.

**Supplementary Table S5. RMSD and sequence identity for each pair of 10 TMs-containing DMT proteins**

|  | Vrg4 | TPT | YddG | PfCRT^IF^ | PfCRT^OC^ |
| --- | --- | --- | --- | --- | --- |
| Vrg4 | — | *13* | *11* | *11* | *11* |
| TPT | **6.3 (4.8)** | — | *10* | *14* | *14* |
| YddG | **5.3 (4.5)** | **7.6 (5.4)** | — | *7* | *7* |
| PfCRT^IF^ | **5.2 (3.0)** | **7.4 (4.3)** | **5.1 (4.9)** | — | — |
| PfCRT^OC^ | **7.0 (5.5)** | **3.0 (2.5)** | **7.4 (6.2)** | **6.2 (4.5)** | — |

Note – Sequence identity (in %) and RMSD (in Å) are written in italics and bold, respectively. RMSD values were computed on Cɑ atoms by including TMs-connecting loops with the *MatchMaker* function implemented in UCSF Chimera. Values in parentheses corresponded to RMSDs without considering TMs-connecting loops.

**Supplementary Table S6. DMT template proteins used to generate PfCRT models by homology modeling.**

|  | **Vrg4 (PDB: 5oge) – inward-facing state** | **TPT protein (PDB: 5y79) – occluded state** |
| --- | --- | --- |
| **DMT subfamily ^a^** | 2.A.7.13  GDP-Mannose:GMP amntiporter (GMA) | 2.A.7.9  Triose-phosphate Transporter (TPT) |
| **Protein length (amino acids)** | 337 | 329 |
| **Sequence covering in structure (amino acids)** | 21-330 | 20-315 |
| **# TMD** | 10 | 10 |
| **% seq. id. with PfCRT ^b^** | 12.7 | 14.5 |
| **Organism** | *Starkeya novella* | *Galdieria sulphuraria* |
| **Chain used for PfCRT modeling** | E | A |
| **Structure resolution (**Å) | 3.22 | 2.2 |
| **In complex with** | — | 3-phosphoglycerate |
| **Method** | X-ray diffraction | X-ray diffraction |
| **Released structure date** | 2017-11-22 | 2017-10-04 |
| **Reference** | Parker & Newstead, Nature, 2017 | Lee *et al.*, Nat. Plants, 2017 |

Note – ^a^ Code and subfamily are based on the Transport Classification Database (TCDB). ^b^ The percentage of sequence identity (% seq. id.) of the given transporter with PfCRT was provided by the Phyre2 server.

**Supplementary Table S7. List of the PfCRT amino acid sites involved in the architecture of the binding pocket based on the predicted PfCRT^OC^ model and the CASTp 3.0 server.**

| **PfCRT**  **3 letters** | **PfCRT**  **1 letter** | **PfCRT position** | **2D Location ^a^** | **Positions associated with drug resistance phenotype ^b^** |
| --- | --- | --- | --- | --- |
| TYR | Y | 68 | TM 1 | - |
| CYS | C | 72 | TM 1 | Chloroquine |
| ASN | N | 75 | TM 1 | Chloroquine |
| LYS | K | 76 | TM 1 | Chloroquine |
| ALA | A | 79 | TM 1 | - |
| HIS | H | 97 | TM 2 | Piperaquine |
| ASN | N | 98 | TM 2 | - |
| CYS | C | 101 | TM 2 | Piperaquine |
| ASP | D | 137 | TM 3 | - |
| SER | S | 140 | TM 3 | - |
| VAL | V | 141 | TM 3 | - |
| ALA | A | 144 | TM 3 | - |
| PHE | F | 145 | TM 3 | Piperaquine |
| LEU | L | 148 | TM 3 | - |
| GLN | Q | 156 | TM 4 | - |
| SER | S | 157 | TM 4 | - |
| VAL | V | 159 | TM 4 | - |
| LEU | L | 160 | TM 4 | - |
| GLN | Q | 161 | TM 4 | - |
| SER | S | 163 | TM 4 | - |
| LEU | L | 217 | TM 6 | - |
| ALA | A | 220 | TM 6 | Chloroquine |
| LEU | L | 221 | TM 6 | - |
| VAL | V | 224 | TM 6 | - |
| CYS | C | 225 | TM 6 | - |
| ASN | N | 228 | TM 6 | - |
| ARG | R | 231 | TM 6 | - |
| GLN | Q | 253 | TM 7 | - |
| LEU | L | 254 | TM 7 | - |
| PHE | F | 322 | TM 8 | - |
| ASN | N | 326 | TM 8 | Chloroquine |
| ASP | D | 329 | TM 8 | - |
| ASN | N | 330 | TM 8 | - |
| THR | T | 333 | TM 8 | - |
| TYR | Y | 345 | TM 9 | - |
| THR | T | 346 | TM 9 | - |
| SER | S | 349 | TM 9 | - |
| CYS | C | 350 | TM 9 | Piperaquine |
| GLN | Q | 352 | TM 9 | Quinine |
| GLY | G | 353 | TM 9 | Piperaquine |
| ILE | I | 356 | TM 9 | Chloroquine |

Note – ^a^ 2D location indicates on which TM the amino acid site is located. ^b^ “ – “ indicates no known relationship with a drug resistance phenotype.

**Supplementary Table S8. PAML results for different structure-based partitionings of the *crt* gene.**

| **Model** | **ℓ** | ***r*_2_** | **κ** | ***ω*** |
| --- | --- | --- | --- | --- |
| **Partitions: cytoplasmic-half (n = 146 codon sites) *versus* vacuolar-half (n = 196 codon sites) sides** | | | | |
| A (homogeneous model) | -7,914.23 | — | 2.34 | 0.091 |
| B (different *r*s) | -7,906.94 | 0.80 | 2.34 | 0.090 |
| C (different *r*s and πs) | -7,922.95 | 0.70 | 2.35 | 0.092 |
| D (different *r*s, κ, and *ω*) | -7,897.08 | 0.89 | κ_1_ = 2.58; κ_2_ = 2.19 | *ω*_1_ = 0.121; *ω*_2_ = 0.070 |
| E (different *r*s, κ, *ω* and πs) | -7,913.51 | 0.78 | κ_1_ = 2.59; κ_2_ = 2.25 | *ω*_1_ = 0.124; *ω*_2_ = 0.072 |
| **Partitions: non-TMs (n = 116 codon sites) *versus* TMs (n = 226 codon sites)** | | | | |
| A (homogeneous model) | -7,914.23 | — | 2.34 | 0.091 |
| B (different *r*s) | -7,885.07 | 0.62 | 2.32 | 0.090 |
| C (different *r*s and πs) | -7,882.14 | 0.85 | 2.27 | 0.090 |
| D (different *r*s, κ, and *ω*) | -7,878.79 | 0.70 | κ_1_ = 2.31; κ_2_ = 2.35 | *ω*_1_ = 0.117; *ω*_2_ = 0.076 |
| E (different *r*s, κ, *ω* and πs) | -7,876.75 | 0.94 | κ_1_ = 2.08; κ_2_ = 2.40 | *ω*_1_ = 0.111; *ω*_2_ = 0.078 |

Note – ℓ, log-likelihood value; *r*_s_, nucleotide substitution rates (*r*_2_ is the nucleotide substitution rate of the second partition, while *r*_1_ is fixed at 1); πs, codon frequencies; κ, transition/transversion rate ratio; *ω*, *d*_N_/*d*_S_ rate ratio; n, number of codon sites; TMs, transmembrane helices; non-TMs, loops connecting two TMs. For the cytoplasm / vacuole partitioning, *r*_1_ corresponds to the nucleotide substitution rate of codon sites located at the cytoplasmic-half side of PfCRT; for the TMs / non-TMs partitioning, *r*_1_ is the nucleotide substitution rate for codon sites located within non-TMs. For the two series of partitioning tests shown here, codon sites were partitioned with the PfCRT^IF^ model and we discarded the poorly conserved N- and C-terminal extremities, both located in the cytoplasm (this alteration was conservative). Similar results were obtained when codon sites were partitioned with the PfCRT^OC^ model (data not shown).

**Supplementary Table S9. List of the 10% most conserved PfCRT amino acid sites using the PfCRT^IF^ model with the FuncPatch server.**

| **Amino acid**  **3 letters** | **Amino acid**  **1 letter** | **PfCRT position** | **2D Location ^a^** | **Involved in ^b^** | |
| --- | --- | --- | --- | --- | --- |
|  |  |  |  | **Cavity** | **Binding pocket** |
| GLY | G | 153 | TM 4 | Yes | No |
| ASN | N | 154 | TM 4 | Yes | No |
| MET | M | 168 | TM 4 | No | No |
| ILE | I | 194 | TM 5 | Yes | No |
| ASN | N | 326 | TM 8 | Yes | Yes |
| ILE | I | 327 | TM 8 | No | No |
| CYS | C | 328 | TM 8 | No | No |
| ASP | D | 329 | TM 8 | Yes | Yes |
| SER | S | 341 | TM 8-TM 9 loop | No | No |
| THR | T | 342 | TM 9 | Yes | No |
| MET | M | 343 | TM 9 | No | No |
| THR | T | 344 | TM 9 | No | No |
| TYR | Y | 345 | TM 9 | Yes | Yes |
| THR | T | 346 | TM 9 | Yes | Yes |
| ILE | I | 347 | TM 9 | No | No |
| VAL | V | 348 | TM 9 | No | No |
| SER | S | 349 | TM 9 | Yes | Yes |
| CYS | C | 350 | TM 9 | Yes | Yes |
| ILE | I | 351 | TM 9 | No | No |
| GLN | Q | 352 | TM 9 | Yes | Yes |
| GLY | G | 353 | TM 9 | Yes | Yes |
| PRO | P | 354 | TM 9 | No | No |
| ALA | A | 355 | TM 9 | No | No |
| ILE | I | 356 | TM 9 | Yes | Yes |
| ALA | A | 357 | TM 9 | Yes | No |
| ILE | I | 358 | TM 9 | No | No |
| ALA | A | 359 | TM 9 | No | No |
| TYR | Y | 360 | TM 9 | Yes | No |
| TYR | Y | 384 | TM 10 | Yes | No |

Note – The 10% most conserved sites of the PfCRT^IF^ model at the *Plasmodium* scale were identified using the FuncPatch server. The results were very similar when we used the PfCRT^OC^ model.

^a^ 2D location indicates on which TM or loop the amino acid site is located.

^b^ For each amino acid site, we indicate whether it is located in the cavity and binding pocket of PfCRT^IF^ and PfCRT^OC^, respectively.

**Supplementary Table S10. Strictly conserved PfCRT amino acid sites involved in the architecture of the binding pocket (using the PfCRT^OC^ model).**

| **Amino acid**  **3 letters** | **Amino acid**  **1 letter** | **PfCRT position** | **2D Location ^a^** | **Positions associated with drug resistance phenotype ^b^** |
| --- | --- | --- | --- | --- |
| TYR | Y | 68 | TM 1 | - |
| ASN | N | 75 | TM 1 | Chloroquine resistance |
| LYS | K | 76 | TM 1 | Chloroquine resistance |
| ASN | N | 98 | TM 2 | - |
| CYS | C | 101 | TM 2 | Piperaquine resistance |
| ASP | D | 137 | TM 3 | - |
| VAL | V | 141 | TM 3 | - |
| LEU | L | 148 | TM 3 | - |
| GLN | Q | 156 | TM 4 | - |
| SER | S | 157 | TM 4 | - |
| GLN | Q | 161 | TM 4 | - |
| LEU | L | 221 | TM 6 | - |
| ARG | R | 231 | TM 6 | - |
| GLN | Q | 253 | TM 7 | - |
| ASN | N | 326 | TM 8 | Chloroquine resistance |
| ASP | D | 329 | TM 8 | - |
| ASN | N | 330 | TM 8 | - |
| TYR | Y | 345 | TM 9 | - |
| THR | T | 346 | TM 9 | - |
| SER | S | 349 | TM 9 | - |
| CYS | C | 350 | TM 9 | Piperaquine resistance |
| GLN | Q | 352 | TM 9 | Quinine resistance |
| GLY | G | 353 | TM 9 | Piperaquine resistance |
| ILE | I | 356 | TM 9 | Chloroquine resistance |

Note – The identification of the strictly conserved amino acid sites of PfCRT was based on a set of 24 different *Plasmodium* species (Supplementary Table S1).

^a^ 2D location indicates the TM on which the amino acid site is located.

^b^ “ – “ indicates no known relationship with a drug resistance phenotype. Of note, the position 76, associated with the key chloroquine resistance mutation K76T, belongs to the strictly conserved amino acid sites and is involved in the architecture of the binding pocket.
